# The Effects and Mechanisms of Water-Soluble Viscosity Modifying Admixtures in the Performance Evolution of Cementitious Materials: A Comprehensive Review

**DOI:** 10.3390/ma19122466

**Published:** 2026-06-09

**Authors:** Lixiao Zhao, Tangzhen Li, Wenlong Wang

**Affiliations:** School of Nuclear Science, Energy and Power Engineering, Shandong University, Jinan 250061, China; zhaolixiao179@163.com (L.Z.);

**Keywords:** water-soluble viscosity-modifying admixtures, cementitious materials, rheological behavior, hydration and microstructure, long-term durability

## Abstract

Water-soluble viscosity-modifying admixtures (VMAs) were initially introduced into cementitious materials to enhance cohesion, stability and resistance to bleeding and segregation. With the development of self-compacting concrete, underwater concrete, grouting materials and 3D-printed cementitious materials, VMAs have become increasingly important for regulating rheological behavior, workability retention, shape retention and construction processability. Recent studies further indicate that VMAs can affect not only fresh-state properties, but also hydration kinetics, early-age microstructure evolution, mechanical performance, transport behavior and long-term durability. This review systematically summarizes the types, action mechanisms, and performance effects of water-soluble VMAs in cementitious materials. Particular emphasis is placed on the relationships among the molecular structure, liquid phase viscosity enhancement, particle adsorption and bridging, polymer-chain entanglement, ion-responsiveness, admixture compatibility, and microstructure evolution. The review shows that the effects of VMAs are not governed solely by admixture type or dosage, but depend strongly on molecular mass, functional groups, substituent composition, charge characteristics, binder chemistry, and the pore solution environment. Finally, current research gaps and future directions are discussed, including quantitative structure–mechanism–performance relationships, applicability in low-carbon binders, service-life prediction, and application-oriented VMA design.

## 1. Introduction

The increasing use of cementitious materials in self-compacting, underwater, grouting, and 3D-printing applications requires a combination of sufficient flowability, high stability, shape retention, and controllable construction windows [1,2,3,4,5,6,7,8,9,10,11]. Therefore, achieving a balance between high flowability and high stability has become a key issue in the mixture design and performance regulation of modern cementitious materials [2,6,7,10,11]. Superplasticizers improve flowability mainly by enhancing particle dispersion. However, at low yield stress and high fluidity, cementitious materials may suffer from insufficient cohesion, reduced aggregate suspension capacity, and compromised stability [1,2,3,4,12,13,14,15,16,17,18]. Consequently, viscosity-modifying admixtures (VMAs) have become important functional admixtures for regulating the rheology and stability of cementitious materials [1,2,3,4]. Among various VMAs, water-soluble VMAs, such as cellulose ethers (CE), polysaccharides, and polyacrylamides (PAMs), have received extensive attention because of their pronounced effects on water retention, rheology, and mixture stability [19,20,21,22,23,24,25,26,27,28,29,30,31,32,33,34,35]. Their actions are not limited to the increasing the liquid-phase viscosity but also involve particle–interface interactions, polymer chain entanglement and network formation, ion-responsive behavior, and interactions with other admixtures [12,13,14,15,16,17,18,25,34,35,36,37,38,39,40,41,42,43,44,45,46,47]. Accordingly, their effects are governed not only by dosage but also by molecular mass, functional groups, substituent type, charge characteristics, and the composition of the cementitious system [17,21,22,23,24,25,27,36,37,38,39].

Research on VMAs in cementitious materials has extended from fresh-state rheology and construction stability to hydration kinetics, early-age microstructural evolution, mechanical performance, and long-term durability [28,30,48,49,50,51,52,53,54,55,56,57,58,59,60,61,62,63,64,65,66,67,68,69,70,71,72,73,74,75,76,77,78,79]. Several reviews have summarized related studies from different perspectives. Khayat et al. reviewed the fundamental functions and engineering applications of VMAs in cementitious materials [2]; Boutouam et al. focused on plant-derived biopolymer-based VMAs in cementitious materials [19]; Xu et al. discussed the effects of VMAs on the rheological properties of cementitious composites [40].Nevertheless, existing reviews have mainly focused on material types, individual properties, or isolated mechanisms. A coherent review is still needed to clarify how the molecular structures of water-soluble VMAs govern their mechanisms of action and thereby affect the fresh-to-hardened performance evolution of cementitious materials.

The effects of VMAs should be considered as a continuous process rather than as isolated property changes. Their molecular structures largely govern their interactions with water molecules, ions, particle surfaces, and hydration products, thereby influencing fresh-state behavior, hydration, microstructural development, mechanical performance, and durability [34,35,36,37,38,39,40,41,42,80,81,82,83,84,85,86,87,88,89,90,91,92,93,94,95,96,97,98,99,100,101,102,103,104,105,106,107,108,109,110,111,112,113,114,115,116,117,118,119,120,121,122,123,124,125,126,127,128,129,130,131,132,133]. Meanwhile, conclusions regarding the same type of VMA are not always consistent across different studies and may even be contradictory. Such inconsistencies are usually associated with the binder composition, the water-to-binder ratio, admixture combination, dosage range, testing age, and evaluation criteria [12,13,14,15,16,17,18,21,22,23,24,25,44,45,101,102,103,104,105,106,107,108,109,110,111,112,113,114,115,116,117,118,119,120,121,122,123,124,125,126,127]. In particular, as VMAs are increasingly combined with polycarboxylate superplasticizers (PCEs), supplementary cementitious materials, defoamers, and other functional components, their action mechanisms and performance responses become more complex [10,11,12,13,14,15,16,17,18,26,27,44,45,46,47,94,134].

Based on these considerations, this review examines the structural characteristics, action mechanisms, and performance-regulating effects of water-soluble VMAs in cementitious materials. Unlike previous reviews that mainly focused on VMA types, individual properties, or specific mechanisms, this paper attempts to establish an integrated analytical framework linking molecular structure, action mechanisms, microstructural evolution, and macroscopic performance. Within this framework, recent progress is synthesized in terms of fresh-state behavior, hydration and early-age microstructure, mechanical performance, and durability. The origins of inconsistent findings in the literature are further analyzed, and mixture design principles, research gaps, and future directions are discussed. This review aims to support the rational selection, precise design, and functional extension of water-soluble VMAs in both conventional and emerging cementitious systems.

To improve the transparency and reproducibility of this review, a structured bibliographic search strategy was adopted. Relevant publications were collected mainly from the Web of Science Core Collection and ScienceDirect. The literature search covered publications available up to April 2026, with an emphasis on cement chemistry, concrete admixtures, rheology, hydration, microstructure, mechanical properties, and the durability of cementitious materials. The search strategy combined terms related to viscosity-modifying admixtures, water-soluble polymers, and cementitious materials. The main search terms included “viscosity-modifying admixture”, “VMA”, “water-soluble polymer”, “cement paste”, “mortar”, “concrete”, “rheology”, “hydration”, “microstructure”, “durability”. Boolean combinations were used, for example: (“viscosity-modifying admixture” OR “VMA” OR “water-soluble polymer”) AND (“cement” OR “cementitious materials” OR “mortar” OR “concrete”) AND (“rheology” OR “hydration” OR “microstructure” OR “mechanical properties” OR “durability”). The inclusion criteria were: (i) studies focusing on water-soluble VMAs or water-soluble polymers used for viscosity modification in cementitious materials; (ii) studies reporting effects on fresh-state behavior, hydration kinetics, microstructural evolution, mechanical properties, or durability-related performance; and (iii) studies providing experimental, theoretical, or simulation-based evidence relevant to VMA mechanisms. The exclusion criteria were: (i) studies focusing only on inorganic viscosity regulators without water-soluble polymeric VMAs; and (ii) studies unrelated to cementitious materials. After the initial search, titles, abstracts, and keywords were screened to remove irrelevant publications. The remaining papers were evaluated based on a full-text reading. Additional references were identified through backward and forward citation tracking of key publications.

## 2. Types, Mechanisms, and Applications of VMAs in Cementitious Materials

### 2.1. Types of VMAs in Cementitious Materials

VMAs used in cementitious materials can be broadly classified, according to their origin, into natural, semi-synthetic, and synthetic types [19,20,26]. Natural VMAs mainly include microbial and plant-derived polysaccharides, such as Welan gum, Guar gum, Diutan gum, and starch-based polymers [19,28,29,30]. These VMAs generally contain long molecular chains and abundant hydrophilic groups, enabling them to improve mixture stability by enhancing water retention and liquid-phase viscosity. Semi-synthetic VMAs are usually obtained by the chemical modification of natural polymers. Cellulose ethers (CEs) are the most representative examples, such as methyl cellulose (MC), hydroxyethyl cellulose (HEC), hydroxyethyl methyl cellulose (HEMC), and hydroxypropyl methyl cellulose (HPMC) [20,24,26,57]. Unlike native cellulose, which has limited water solubility, CEs can be designed to dissolve or swell in aqueous pore solutions and provide effective viscosity modification. Their molecular mass, substituent group, and degree of substitution can be adjusted, allowing them to provide multiple functions, including viscosity enhancement, water retention, and workability regulation [20,21,22,23,24,25,27,36,37,38,39]. Synthetic VMAs have greater molecular design flexibility and mainly include polyacrylamide and its derivatives, polyacrylic acid-based polymers, and some vinyl polymers [26,27,32,33,34,35]. Their chain length, charge density, and functional groups can be tailored to regulate water retention, adsorption behavior, and rheological responses.

Besides origin, VMAs can also be classified according to their charge characteristics [20,28,29,30,31,32,33]. Nonionic VMAs are typically represented by CEs, neutral polyethers, and some neutral polysaccharides. Their effects are mainly associated with liquid-phase viscosity enhancement, water retention, and polymer chain entanglement, whereas their electrostatic interactions with particle surfaces are relatively weak [20,28,29,30]. Ionic VMAs contain ionizable or charged groups, such as carboxylate groups, can interact with Ca^2+^ and particle surfaces in highly alkaline pore solutions. Representative examples include carboxymethyl chitosan, polyacrylic acid, and sodium alginate [31,32,33,82]. It should be noted that VMA classification should not remain at the simplified categorical level of “natural/synthetic” or “ionic/nonionic”. Molecular parameters are more directly related to their mechanisms and performance. VMA classification is therefore useful for identifying possible dominant mechanisms, but it cannot directly predict engineering performance.

### 2.2. Fundamental Action Mechanisms of VMAs in Cementitious Materials

The effects of VMAs arise from the coupled regulation of the liquid-phase, particle interfaces, polymer networks, and pore solution chemistry. Based on existing studies, their fundamental mechanisms can be summarized as liquid-phase viscosity enhancement and water regulation, adsorption and bridging at particle surfaces, polymer chain entanglement and network formation, and ion-responsive behavior together with admixture interactions [1,2,3,4,12,13,14,15,16,17,18,40,41,42,43].

Liquid-phase viscosity enhancement and water regulation represent the most fundamental modes of action of VMAs [1,2,3,4]. Water-soluble VMA molecules can form hydrogen bonds or other interactions with water molecules, causing part of the free water to be adsorbed. This increases the pore solution viscosity and reduces the water migration rate [21,22,23,24,25]. Macroscopically, this process leads to higher plastic viscosity, improved water retention, reduced bleeding and segregation, and enhanced mixture stability.

Adsorption and bridging at particle surfaces are important mechanisms through which VMAs modify interparticle interactions and structural build-up behavior [1,2,3,4,36,41,42]. Some VMAs can adsorb onto cement particles or early hydration products, altering the interfacial state of particles and increasing the resistance to their relative movement [25,36,41,42,43]. When polymer chains interact simultaneously with multiple particles or hydration products, bridging structures may form, thereby strengthening interparticle interactions and modifying the yield stress and structural integrity of the paste [29,31,34,56]. This mechanism is often more pronounced for VMAs containing charged groups, because they can interact with Ca^2+^ and particle surfaces through ionic complexation or bridging, promoting flocculation and network formation [31,32,33,34,35,82,83].

Polymer chain entanglement and network formation provide another important basis for improving cohesion, thixotropy, and shape retention capacity [1,2,3,4,43,51,52,53,54,55,56,57,58]. When VMAs have a relatively high molecular mass or are incorporated at a relatively high dosage, polymer chains are more prone to entanglement and may form continuous or semi-continuous network structures [56,57]. These structures increase flow resistance in the liquid phase and restrict the migration of fine particles, bubbles, and free water, thereby enhancing cohesiveness, static stability, and segregation resistance. However, an excessively strong polymer network structure may also increase pumping resistance, accelerate flowability loss, and shorten the construction window.

The ionic environment of the pore solution and the combined use of multiple admixtures further determine the actual performance of VMAs in cementitious materials [12,13,14,15,16,17,18,34,35,36,44,45,46,47,81,82,83]. Cement pore solutions are highly alkaline and rich in Ca^2+^, which can affect the molecular conformation, adsorption behavior, and effective concentration of VMAs [12,13,14,15,16,17,18,34,35,36,81,82,83]. For polymers containing charged groups, Ca^2+^ may induce complexation, ionic bridging, or local gelation [12,13,14,15,16,17,18,34,35,82,83]. For neutral polar groups, such as amide and ether groups, the interactions are more often associated with hydrogen bonding and adsorption. Meanwhile, VMAs are commonly used together with PCEs, defoamers, and supplementary cementitious materials. Their final performance therefore often depends on the synergistic or competitive interactions among multiple components [12,13,14,15,16,17,18,44,45,46,47].

### 2.3. Typical Application Scenarios of VMAs in Cementitious Materials

VMAs are used to balance flowability and stability, but their dominant functions vary among applications [2,6,7]. They have been widely applied in underwater anti-washout concrete, self-compacting concrete, grouting and repair materials, lightweight or functional composites, and 3D-printed cementitious materials [2,6,7,8,9,10,11,51,52,53,54,55,56,57,58].

Underwater anti-washout concrete represents one of the earliest important applications of VMAs. By improving paste cohesion and washout resistance, VMAs reduce paste loss and aggregate segregation during underwater placement, thereby meeting the requirements of underwater casting, marine engineering, and deep foundation construction [2]. In self-compacting concrete and other highly flowable concretes, VMAs increase paste viscosity and aggregate suspension capacity, thereby improving segregation resistance and broadening the mixture design window [6,7]. These systems typically rely on superplasticizers to achieve high flowability, whereas VMAs compensate for the insufficient stability caused by low yield stress. Therefore, the compatibility and synergistic effects between superplasticizers and VMAs play a critical role in determining material performance [6,7,12,13,14,15,16,17,18,44,45]. In grouting, repair, and rendering materials, VMAs mainly provide water retention, bleeding resistance, improved construction adhesion, and enhanced interfacial quality [5,20,24,57]. In lightweight concrete and composites containing special functional particles, VMAs can suppress particle floating or settlement, improve component homogeneity, and regulate early-age water loss [8,9,135].

With the development of digital construction technologies, 3D-printed cementitious materials have become an important emerging application field for VMAs [10,11]. Unlike conventionally cast materials, 3D-printing materials require sufficient flowability during pumping and extrusion, while rapidly rebuilding their internal microstructure after deposition to support subsequent layer stacking [10,11,51,52]. Therefore, the role of VMAs in such systems is no longer limited to viscosity enhancement; rather, they contribute to the balance among pumpability, extrudability, buildability, and the printable window [11,51,52,53,54,55,56,57,58]. In low-carbon binders such as limestone calcined clay cement (LC^3^), and in 3D-printed ultra-high performance-concrete (UHPC) systems, the coupling among VMAs, superplasticizers, fine particles, and particle packing becomes more prominent. Their mechanisms and design principles therefore need to be further analyzed in relation to the specific material composition [55,66,67,76,78,94].

## 3. Effects of VMAs on Fresh-State Behavior of Cementitious Materials

### 3.1. Rheological Behavior and Structural Build-Up

Rheology is central to evaluating the fresh-state regulation of VMAs. Yield stress and plastic viscosity describe the resistance to flow initiation and sustained flow, respectively. In cementitious materials, yield stress should be further distinguished as dynamic yield stress and static yield stress. Dynamic yield stress is usually determined under continuous shearing or after flow has been initiated and is more closely related to flowability, pumping, and extrusion. Static yield stress refers to the stress required to initiate flow after the material has remained at rest for a certain period, and is strongly associated with structural build-up, thixotropic recovery, shape retention, and buildability. Therefore, the effects of VMAs on yield stress should be interpreted according to the testing protocol and the targeted performance requirement. In general, most VMAs increase the plastic viscosity of cementitious pastes and, under certain conditions, may also increase static or dynamic yield stress. However, these effects are not linear and depend strongly on VMA type, molecular weight, functional groups, dosage, binder composition, and pore solution chemistry [21,22,23,24,25,27,59,60].

**Figure 1:**
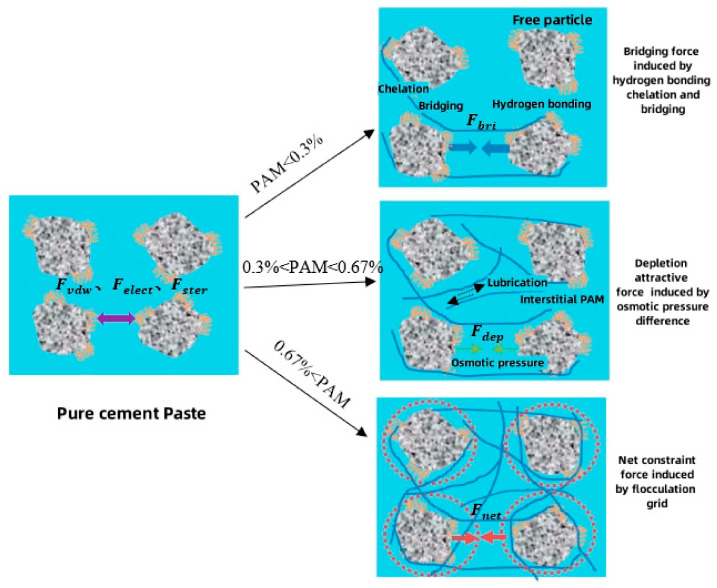
Mechanistic model for the effect of PAM content on flowability, reprinted from [136], the blue line donates PAM, Copyright (2025), with permission from Creative Commons Attribution (CC BY 4.0) license.

The effect of VMAs on the dynamic yield stress is often non-monotonic and VMA system-dependent. Some polymeric ethoxylated derivatives, natural polysaccharides, and starch derivatives generally increase dynamic yield stress [27,30,61], whereas VMAs such as Diutan gum, HEMC, HPG, HPMC, and alkyl-chain-modified HPG may exhibit different trends, such as an initial increase followed by a decrease or the opposite trend [28,30,48,49,50]. These differences indicate that the dynamic yield stress is governed by the balance among particle flocculation, polymer adsorption and bridging, steric hindrance, and polymer network formation. Adsorptive or ionic VMAs can enhance interparticle interactions through surface adsorption and bridging, thereby strengthening flocculated structures and increasing the dynamic yield stress [29,31,34,56]. Weakly adsorbing or nonionic VMAs mainly increase shear resistance by enhancing the liquid-phase viscosity and promoting chain entanglement or network formation [65]. At higher dosages, however, steric hindrance and changes in flocculation structure may weaken direct particle contacts, leading to a reduction in yield stress [63,64]. At further increasing dosages, entanglement within the polymer molecular network may lead to a higher dynamic yield stress. The mechanistic model proposed by Lu et al. [136] for the effect of the PAM content on flowability provides a clear explanation of how VMA dosage influences the yield stress, as shown in Figure 1.

Plastic viscosity is generally more sensitive to VMAs than yield stress. In most cases, VMAs increase the flow resistance of cementitious pastes by increasing the liquid-phase viscosity and polymer chain entanglement; therefore, an increase in plastic viscosity is more commonly observed [27,59,60]. Nevertheless, plastic viscosity does not necessarily increase monotonically with VMA dosage. Some studies have shown that VMAs may reduce paste viscosity by improving the lubrication state between particles or modifying the flocculated structure [50]. Therefore, changes in plastic viscosity reflect not only the liquid-phase viscosity-enhancing effect, but also the particle dispersion state and the flocculation structure.

**Figure 2:**
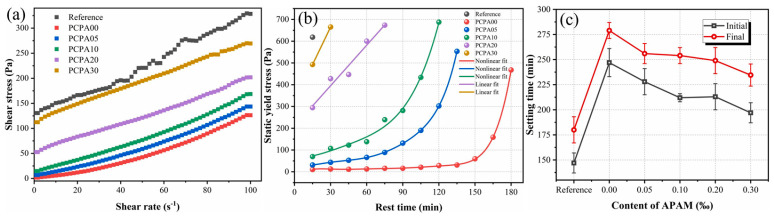
Effects of combined PCE and APAM incorporation on the fresh-state properties of cement paste: (**a**) dynamic yield stress, (**b**) static yield stress, (**c**) setting time, reprinted from [137], Copyright (2024), with permission from CC BY 4.0 license.

In highly flowable cementitious materials, VMAs are commonly combined with superplasticizers, and their interactions strongly affect rheology and structural build-up. Superplasticizers the reduce dynamic yield stress and improve flowability mainly through particle dispersion, whereas VMAs enhance stability through liquid-phase viscosity enhancement, water retention, and particle bridging [44,45]. Zou et al. [137] incorporated PCEs and anionic PAM (APAM) into cement paste and found that increasing the APAM dosage increased the dynamic and static yield stress of the paste, accelerated structural build-up, and shortened the setting time. The static yield stress, dynamic yield stress, and setting time are shown in Figure 2. However, their effects are not simply complementary. Competitive adsorption or incompatibility may occur between specific VMAs and superplasticizers. For example, abnormal thickening may occur between CEs and certain sulfonate-based superplasticizers [12], while HPMC and naphthalene-based superplasticizers may even form gels under highly alkaline conditions [13]. For PCEs, competitive adsorption between CEs and PCEs, together with Ca^2+^-mediated interactions, can reduce the dispersing efficiency of PCEs [14,15,16,17]. The effects of ionic VMAs, such as sodium alginate, sodium gluconate, and polyacrylic acid, are closely related to molecular mass, charge density, and dosage. At low dosages, they may contribute to dispersion or water regulation, whereas at high dosages they are more likely to increase viscosity and cause flowability loss [18,32,33].

Such interactions can further affect structural build-up. Studies have shown that, under comparable initial flowability, competitive adsorption between VMAs and PCEs can significantly modify the structural build-up of UHPC mortars. Compared with CEs and latex polymers, anionic Welan gum and Diutan gum, which possess a stronger adsorption capacity, increase the demand for superplasticizer dosage but can still more effectively enhance the structural build-up rate [67]. Similarly, anionic biopolymers are more likely to weaken the dispersing effect of superplasticizers through competitive adsorption and to promote flocculated networks, thereby improving elasticity, static yield stress, and structural build-up capacity. In contrast, the strengthening effect of nonionic biopolymers is usually weaker and more sensitive to dosage [68]. Therefore, the influence of VMAs on rheological behavior should not be evaluated only by instantaneous yield stress and plastic viscosity. The structural build-up rate, thixotropic recovery, and shear history should also be considered.

### 3.2. Workability Retention and Fresh-State Stability

Workability retention and fresh-state stability are also key indicators for evaluating the effects of VMAs. Workability retention reflects the time-dependent preservation of flowability, whereas fresh-state stability refers to the resistance to bleeding, segregation, and particle migration. For highly flowable cementitious materials, low yield stress is beneficial for flow and filling capacity, but it can also increase the risk of bleeding, segregation, and a reduced aggregate suspension capacity. By improving cohesion, VMAs can reduce bleeding and segregation, thereby improving mixture homogeneity [2,71,72].

The effect of VMAs on workability is not inherently adverse. Although VMAs usually increase the liquid-phase viscosity and reduce initial flowability, appropriate dosages may improve flow retention and construction stability by enhancing paste homogeneity, increasing water retention, and delaying water loss [71,72,73,74,75]. For example, the molecular mass of polyacrylamide affects not only the plastic viscosity of cementitious pastes but also the workability of the material by modifying electrostatic repulsion and the bridging ability between particles [73]. Polysaccharide-based VMAs may increase slump flow and enhance flow retention when combined with a suitable type and dosage of a superplasticizer [74]. Meanwhile, the matching between VMA type and superplasticizer type plays an important role in controlling the workability of high-performance self-compacting concrete. Even when their chemical compositions are similar, superplasticizers and VMAs from different sources should not be simply substituted for one another [75].

Stability control becomes particularly important in composites with large density differences among components. For example, in concrete containing lightweight expanded polystyrene beads, hydroxypropyl cellulose ether (HPC) can significantly suppress segregation and anisotropy, although it also reduces flowability [135]. This result reflects a fundamental trade-off in VMA-regulated fresh-state behavior: improved stability is often accompanied by reduced flowability. Therefore, the VMA dosage should be optimized according to the required balance among flowability, segregation resistance, flow retention, and the construction window.

### 3.3. Printability and Shape Retention

**Figure 3:**
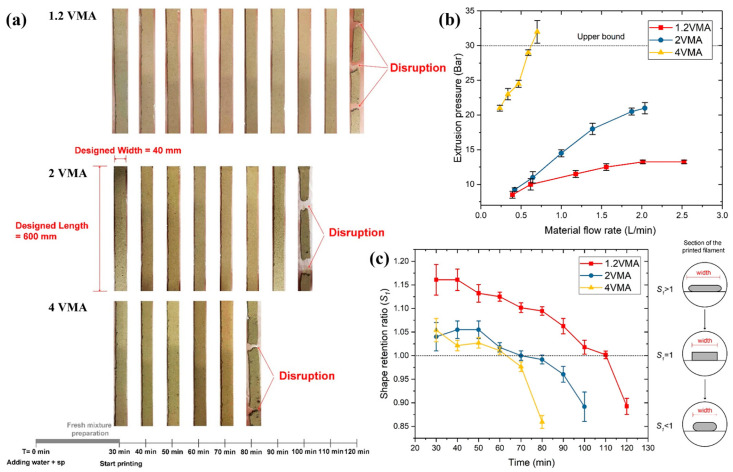
Effect of HPMC incorporation on the printability of limestone calcined clay-based cementitious materials: (**a**) open time, (**b**) extrusion pressure, (**c**) shape retention ratio, reprinted from [76], Copyright (2024), with permission from CC BY 4.0 license.

With the development of 3D-printed cementitious materials, increasing attention has been paid to the role of VMAs in regulating printability. Printability generally includes pumpability, extrudability, and buildability. For 3D-printed cementitious materials, the distinction between dynamic and static yield stress is particularly important. The dynamic yield stress, together with the plastic viscosity, mainly controls flow resistance during pumping and extrusion, whereas the static yield stress, thixotropy, and the structural build-up rate determine shape retention after deposition, buildability, and the load-bearing capacity between layers [51,52,55,76]. Therefore, compared with conventionally cast materials, 3D-printed cementitious materials impose more complex requirements on VMAs: the material must maintain sufficient flowability under shear while rapidly rebuilding its structure after shear removal. Chen et al. incorporated HPMC into 3D-printed limestone calcined clay-based cementitious materials and found that a high HPMC dosage adversely affected printability. Increasing the dosage increased the extrusion pressure, shortened the open time, and reduced the shape retention ratio [76], as shown in Figure 3.

In emerging materials such as 3D-printed UHPC, superplasticizers and VMAs often show both synergistic and competing effects. Superplasticizers improve flowability and extrudability through lubrication and dispersion, but they may weaken water retention and post-deposition shape stability. In contrast, VMAs improve buildability by enhancing cohesion and structural stability, although this is often achieved at the expense of partial flowability [55]. Therefore, a refined balance among pumpability, extrudability, shape retention, and interlayer stacking stability is required when these admixtures are used together [55,66].

Existing studies have shown that MC can increase both the initial bulk yield stress and shear yield stress, thereby improving the shape stability of extruded materials; however, it also increases the extrusion pressure and reduces flowability [52]. Xiao et al. proposed introducing HPMC through a secondary mixing step before printing, which significantly increased the static yield stress while exerting a relatively minor effect on the dynamic yield stress. This strategy is therefore beneficial for balancing buildability and extrudability [51]. Studies on thixotropy have also shown that VMAs generally improve structural recovery, but their effectiveness is closely related to the VMA molecular structure and the superplasticizer type [53,54]. These results indicate that the key role of VMAs in 3D printing is not merely to increase the apparent viscosity, but to regulate structural recovery after shear removal and optimize the printable window.

The applicability of different VMAs in 3D-printed materials varies considerably. MC can improve deformability and continuity during extrusion [77]. Compared with HPMC, HEC exhibits a more pronounced ability to regulate the open time in 3D-printing of LC^3^ systems. It can significantly prolong the initial setting time and the printability window, and it can act together with silica fume to balance the open time and buildability [78]. HPMC can increase the static yield stress and extrusion stability, but its effectiveness is influenced by the paste volume fraction, the aggregate volume fraction, and admixture combination strategy [79]. For polyacrylamide-based VMAs, their relatively high elastic modulus is beneficial for limiting the deformation of deposited layers [55,56,57]. However, Liu et al. found that when APAM was used to partially replace HPMC, an appropriate replacement ratio improved buildability, whereas a higher replacement ratio led to reduced shape stability [58]. Therefore, VMA regulation of printability is essentially an optimization of flowability, structural recovery, deposition stability, and the printable window. Its effectiveness is highly dependent on the binder composition, particle gradation, admixture combination, and dosage design.

Overall, the fresh-state effects of VMAs should be interpreted as the result of competing mechanisms rather than as a direct consequence of viscosity enhancement. Liquid-phase viscosity enhancing mainly increases the plastic viscosity and stability, whereas adsorption, bridging, steric hindrance, and VMA–superplasticizer interactions determine whether the yield stress and structural build-up increase or decrease. This explains why the same type of VMA may produce different rheological responses in different binder systems.

## 4. Effects of VMAs on Hydration and Early-Age Microstructure Evolution of Cementitious Materials

### 4.1. Hydration Kinetics

**Figure 4:**
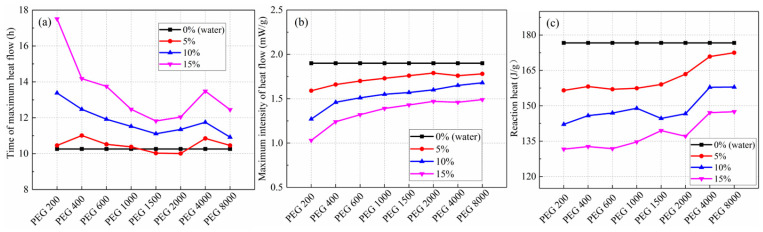
The delaying effect of PEG with different molecular mass on the cement hydration; (**a**) time of maximum heat flow, (**b**) maximum heat flow, (**c**) maximum 72 h cumulative reaction heat, reprinted from [102], Copyright (2021), with permission from Elsevier.

Many water-soluble VMAs tend to retard early cement hydration, as indicated by a prolonged induction period, a delayed or reduced main heat-flow peak, and a lower early cumulative heat release [36,80,81]. As shown in Figure 4, polyethylene glycol (PEG) with a different molecular mass exhibits a clear retarding effect on cement hydration [102]. Such phenomena have also been reported for CEs, polyacrylamide, and other VMAs [34,35,36,81,102,138]. The retardation is generally associated with restricted ion migration, modified particle surface states, and the disturbed nucleation and growth of hydration products. Possible mechanisms include hindered diffusion, adsorption on particle or hydration product surfaces, the formation of protective layers, and nucleation control [89,90,91,92].

In CE-containing systems, early studies attributed hydration retardation mainly to the increased viscosity of the liquid phase, which reduces ion mobility and inhibits clinker phase dissolution and hydration product precipitation [92]. Subsequent studies, however, have shown that the viscosity-induced diffusion alone is not the dominant controlling mechanism [37]. Compared with molecular mass, the substituent group of CEs plays a more critical role in hydration retardation [36,37,38,39]. For example, the retarding effect of different CEs is generally reported to follow the order HEC > HEMC/HPMC > MC, with HEC showing the strongest effect and MC the weakest, while HEMC and HPMC exhibit intermediate behavior [38]. CEs with a lower methyl content, lower hydroxyethyl content, and lower molecular mass often show stronger retarding effects [36,37,38,39]. These findings indicate that CE-induced hydration retardation is governed more by molecular structure and surface interactions than by macroscopic viscosity alone.

From the perspective of mineral phase hydration, C_3_S and C_3_A are two key phases for understanding VMA-induced retardation because they strongly influence early heat evolution, setting, and strength development [93]. For C_3_S hydration, CEs exhibit a relatively weak adsorption on C_3_S itself but stronger adsorption on the surfaces of calcium silicate hydrate (C-S-H) and calcium hydroxide (CH). Therefore, their direct inhibition of C_3_S dissolution is limited, whereas they can significantly modify the precipitation kinetics of C-S-H and CH [41,42]. Specifically, CEs can slow the heterogeneous nucleation of C-S-H on C_3_S surfaces, reduce the number of initial nucleation sites, and increase the critical supersaturation required for CH precipitation, thereby delaying early hydration [42]. For C_3_A hydration, the CE substituent groups are more important controlling factors than the molecular mass. Their adsorption on calcium hydroaluminates surfaces is stronger than that on ettringite, while the presence of gypsum can weaken their inhibitory effect on C_3_A dissolution [36].

The effect of VMAs on hydration kinetics also depends strongly on the binder system. In OPC-LC^2^ systems, a low dosage of methyl hydroxyethyl cellulose (MHEC) can improve water retention and yield stress, but it has a limited influence on the hydration kinetics of mixtures with a high LC^2^ content. When the dosage is further increased, its retarding effects on the calcium aluminosulfate formation (second peak) become more pronounced [94]. The same study also suggested that the additional sources of Ca^2+^ and aluminate phases in LC^2^ could partially mitigate the adverse effect of MHEC on hydration [94]. This indicates that the influence of VMAs on hydration is not determined solely by the intrinsic properties of the polymer, but is closely related to binder mineralogy, pore solution chemistry, and the types of reaction products formed.

In addition to CEs, other VMAs also exhibit significant effects on hydration kinetics. Hydroxypropyl Guar gum can reduce the growth rate of hydration products by adsorbing onto their surfaces, and its retarding effect is influenced by the steric hindrance of the substituent groups [81]. Welan gum prolongs the induction period and suppresses the early hydration of C_3_S and the secondary hydration of aluminate phases, although its effect on cumulative heat release is relatively limited [80]. Polyacrylamide-based VMAs show distinct charge-dependent effects: anionic PAM generally markedly retards hydration and reduces the cumulative heat release, whereas cationic and nonionic PAM have weaker effects on the induction period and may even increase the maximum heat flow rate and cumulative heat release [34,35,82]. Therefore, the differences among VMAs in hydration kinetics essentially arise from differences in functional group charge characteristics, adsorption behavior, and interaction modes with particle surfaces and pore solution ions.

### 4.2. Hydration Products

**Figure 5:**
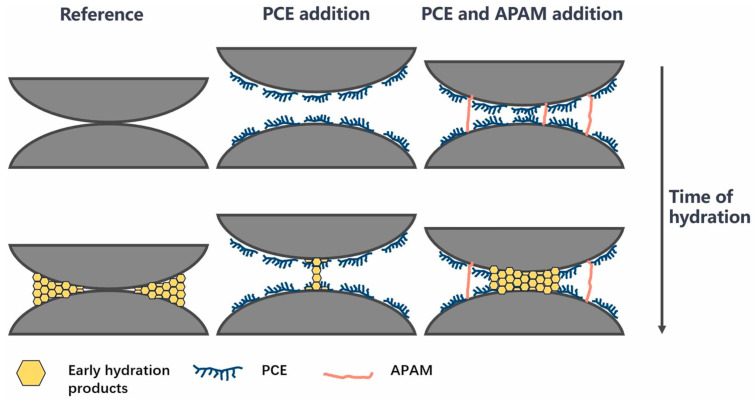
Schematic representation of the mechanisms of synergistic influence of PCE and APAM on cement paste hydration, reprinted from [137], Copyright (2024), with permission from CC BY 4.0 license.

VMAs affect hydration products mainly by modifying their formation rate, content, morphology, and distribution. Existing studies indicate that VMAs usually do not induce new major hydration products. Instead, they regulate the precipitation kinetics, product content, crystal morphology, and distribution of existing hydration products such as CH and C-S-H [80,83,84,85,86,87,88]. Thus, VMAs primarily reshape the formation process of hydration products. Zou et al. suggested that the combined incorporation of PCE and APAM in cement paste promotes the formation of hydration products more effectively than PCE alone, as illustrated by the mechanism shown in Figure 5 [137].

Reported trends for CH are not entirely consistent. Some studies have found that appropriate dosage of VMAs such as HPMC may increase the CH content in the matrix, which is usually attributed to enhanced water retention and improved hydration [84,85]. In contrast, other studies have shown that CEs can reduce the CH content, possibly because they adsorb on CH nucleation sites and inhibit crystal growth, or because they introduce certain uncertainties during thermal analysis [86,87,88]. Although the reported trends vary, most studies agree that VMAs significantly affect the precipitation kinetics and crystal morphology of CH [83,84,88,95]. For example, PAM can reduce the size of CH crystals and decrease their content in the interfacial transition zone [83]. MC can induce CH to form layered stacking arrangements and promote the formation of polymer films between crystals [88]. Figueiredo et al. found that, after the incorporation of HPMC, CH crystals more frequently precipitated on the inner walls of pores rather than being uniformly dispersed within the cement paste matrix. This abnormal distribution was considered to be associated with VMA-induced changes in the local nucleation environment and liquid-phase viscosity [84]. Recent studies have further shown that, with an increasing PAM dosage, both the size and orientation index of CH crystals in the hardened paste decrease, and CH gradually transforms from typical large layered hexagonal crystals into smaller microcrystals with eroded edges [99].

**Figure 6:**
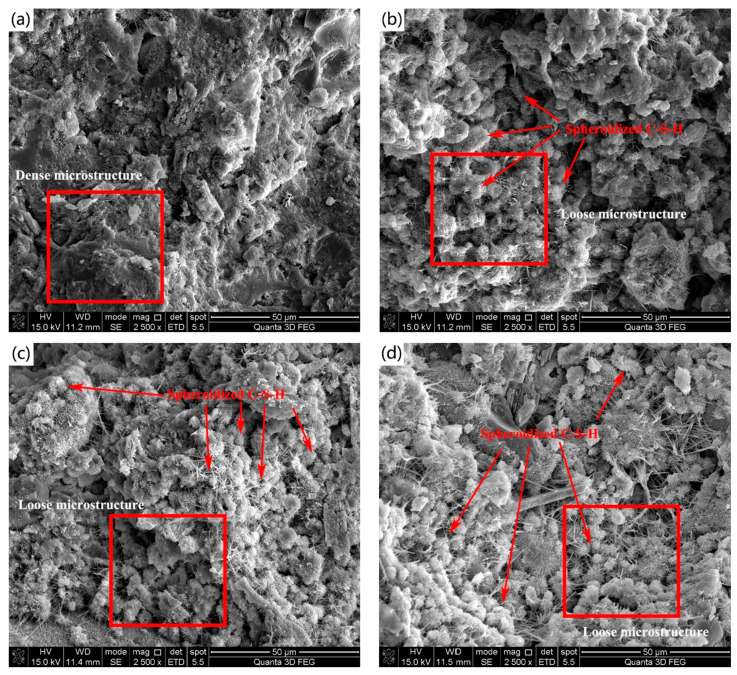
SEM images of the mortars mixed without and with 15% different molecular mass PEG at 28 d (2500×): (**a**) without PEG; (**b**) with PEG600; (**c**) with PEG1500; (**d**) with PEG8000, reprinted from [139], Copyright (2021), with permission from Elsevier.

In addition to CH, VMAs can affect the morphology and packing mode of C-S-H. PEG can self-aggregate to form micellar structures, which may serve as templates for C-S-H growth and promote the formation of spherical C-S-H [96,97]. Molecular dynamics simulations further suggest that PEG may shorten the main-chain length of C-S-H and reduce its packing density [98]. In mortar systems, PEG also modifies the morphology of C-S-H, making it more spherical, reducing the particle size, and leading to a looser packing structure [139], as shown in Figure 6. These results indicate that the interactions between VMAs and hydration products are not limited to retarding or promoting specific reactions. VMAs may also alter the microscopic morphology and spatial organization of hydration products by regulating the local nucleation environment and product aggregation behavior.

### 4.3. Early-Age Microstructure Evolution

By altering hydration kinetics and product formation, VMAs further affect early-age pore structure, organic–inorganic interfaces, and organic phases distribution. These effects are not merely consequences of hydration retardation, but arise from the coupling among hydration reactions, polymer action, and pore structure formation [83,84,88,98,99]. VMAs can influence the spatial organization of hydration products by modifying the nucleation, precipitation, and solid phase filling processes. Meanwhile, they can regulate the organic phase distribution and pore structure formation through water retention, chain entanglement, and interfacial interactions.

The effects of VMAs on the early-age microstructure are often dual. An appropriate dosage may promote later hydration through water retention and internal curing effects, leading to a denser and more homogeneous local structure [84,85]. However, VMAs may also increase the porosity, increase the proportion of capillary pores, or loosen the microstructure due to hydration retardation, air entrainment, or the altered packing of hydration products [86,87,88]. Therefore, the final influence of VMAs on the early-age microstructure depends on the competition between water retention-induced hydration promotion and local densification on the one hand, and structural loosening caused by retardation, air entrainment, and altered product packing on the other.

**Figure 7:**
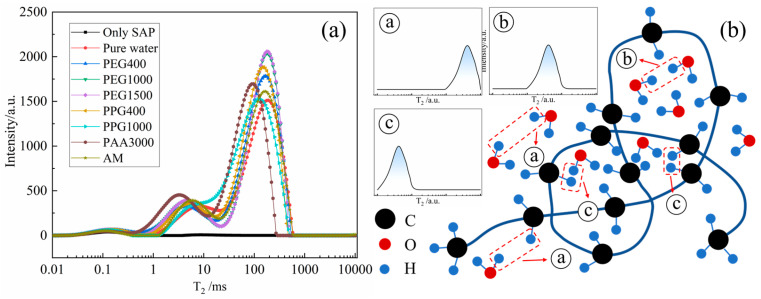
The phenomenon of the leftward shift in the pore size distribution measured by LF-NMR due to the addition of the VMAs (**a**), and the mechanism (**b**); in (**b**), ⓐ denotes the most distant hydrogen atom, ⓑ denotes the medium distance hydrogen atom, and ⓒ denotes the closest hydrogen atom, reprinted from [100], Copyright (2024), with permission from Elsevier.

For VMA-containing systems, low-field nuclear magnetic resonance (LF-NMR) results should be interpreted with caution. Hydrogen bonding between water-soluble polymers and water molecules can alter relaxation behavior, leading to overestimated porosity and an apparent shift in the pore size distribution toward smaller pores [100], as shown in Figure 7. Therefore, LF-NMR results should be corrected or cross-validated with complementary methods before being directly interpreted as real pore-structure changes.

In addition to pore structure changes, the formation of polymer films or network-like structures among hydration products also indicates that VMAs are not merely early-stage solution modifiers, but may directly participate as organic phases in the spatial organization of inorganic hydration products [88,98]. Knapen et al. showed that water-soluble VMAs can form film-like structures during hydration and cover the surfaces of hydration products [88]. Combined with the regulatory effects of PEG on the morphology and packing mode of C-S-H [98,139], these findings suggest that the influence of VMAs on the early-age microstructure is not limited to a single dimension of increasing or decreasing porosity. Instead, it involves the coordinated reconstruction of hydration products, pore structure, and organic phases.

Overall, the regulation of hydration products by VMAs can be summarized at three levels: modifying the precipitation kinetics of products such as CH and C-S-H, affecting crystal size, morphology, and orientation; and reshaping the distribution of hydration products in matrix pores and interfacial regions.

## 5. Effects of VMAs on Hardened-State Mechanical Properties of Cementitious Materials

### 5.1. Compressive Strength

The influence of VMAs on the compressive strength is often unfavorable or only marginally beneficial, depending on VMA type, dosage, molecular structure, water-to-binder ratio, and binder composition [35,101,102,103,104,105]. A relatively consistent trend can be identified from existing studies: when hydration retardation, air entrainment, and pore structure coarsening dominate, the compressive strength usually decreases; when water retention-induced hydration promotion prevails at low VMA dosages, the strength loss may be mitigated, and a slight strength improvement may even occur [67,101,102].

The adverse effect of CE-based VMAs on compressive strength is particularly common. Previous studies have shown that CEs often increase the proportion of capillary pores, or delay early hydration, thereby reducing the compressive strength of hardened cementitious materials [103,104]. Even when the air content of the modified mortars is controlled by defoamers to a level close to that of the reference mixture, the compressive strength of HEMC-modified mortars remains lower than that of the control group. This indicates that the strength loss is not caused solely by air entrainment; the hydration retardation and pore structure evolution also play important roles [104]. Similarly, in sulfoaluminate cement mortars, the compressive strength generally decreases with an increasing CE dosage, while the substituent type and the degree of substitution further affect the magnitude of the strength reduction [105].

The influence of PAM-based VMAs on compressive strength depends strongly on their charge characteristics. Yuan et al. compared different ionic PAMs and found that anionic PAM caused the most pronounced reduction in compressive strength, followed by cationic PAM, whereas nonionic PAM showed relatively small differences from the control group [35]. This behavior is usually attributed to differences in pore structure coarsening and the air-entraining effects of different PAMs, with nonionic PAM exerting a comparatively weaker adverse influence on the pore structure. In systems containing multiple admixtures, the synergistic or competitive interactions between VMAs and superplasticizers can further alter the early strength development, making the compressive strength response more complex [67,68].

**Figure 8:**
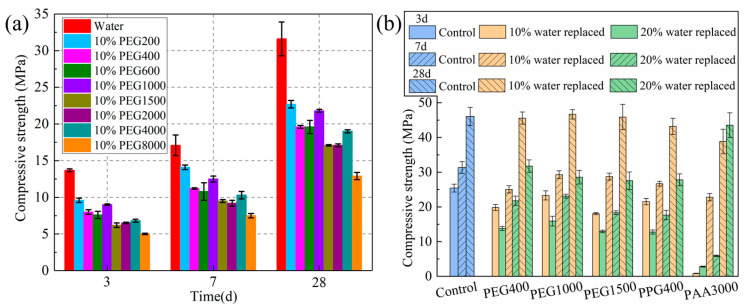
Effect of polyether-based VMA addition on the compressive strength of mortar: (**a**) direct addition, reprinted from [102], Copyright (2021), with permission from Elsevier; (**b**) replacement of mixing water, reprinted from [131], Copyright (2024), with permission from Elsevier.

Polyether-based VMAs, such as PEG, influence the compressive strength of cementitious materials depending on their molecular mass and addition method. When 10% PEG was added directly, the compressive strength of mortar specimens from 3 d to 28 d was lower than that of the control group. Moreover, as the molecular mass increased from 200 to 8000, the compressive strength decreased monotonically [102], as shown in Figure 8a. At the microscale, this reduction may be attributed to the possible thinning of the Ca(OH)_2_ crystals formed during hydration following PEG addition, which weakens the mortar matrix [140,141]. In addition, long-chain PEG may insert into the pores of C-S-H particles, resulting in low-density packing of C-S-H [98] and thereby adversely affecting the strength. At the macroscale, PEG addition may introduce additional air bubbles or the entrapment of bubbles, producing internal defects that also reduce the mortar strength [101]. When 10% PEG was added as a replacement for mixing water, it had little effect on the 7 d compressive strength of the mortar. The optimal compressive strength was obtained with PEG having a molecular mass of 1000, and its 3 d strength was already close to that of the control group. This may be because the reduction in the effective water-to-cement ratio helps decrease matrix porosity, whereas VMA incorporation tends to increase porosity. The nearly unchanged compressive strength therefore reflects the competing effects of the reduced water-to-cement ratio and VMA addition on the matrix porosity [131], as shown in Figure 8b.

Nevertheless, VMAs do not necessarily reduce compressive strength. At low dosages or in suitable binder systems, some VMAs can improve water retention, reduce early-age water loss, and provide an internal curing effect, thereby promoting later hydration and compensating for the early strength loss [61,80]. Therefore, the influence of VMAs on compressive strength should be understood as the result of the competition between water retention-induced hydration promotion and the negative effects associated with hydration retardation, air entrainment, and pore structure coarsening.

### 5.2. Flexural and Tensile Behavior

Compared with compressive strength, flexural strength, tensile behavior, and toughness are more likely to benefit from VMA-induced interfacial bonding and crack-bridging effects [34,35,106,107,108,109,110]. These properties depend not only on the matrix compactness but also on interfacial bonding, crack propagation paths, and energy dissipation capacity. Although VMAs may reduce compressive strength due to hydration retardation, air entrainment, or pore-structure coarsening, the polymer films, particle-bridging structures, and organic–inorganic interfacial interactions formed in VMA-containing cementitious materials may improve the resistance to crack initiation and propagation [83,88,106,107,108,109,110].

**Figure 9:**
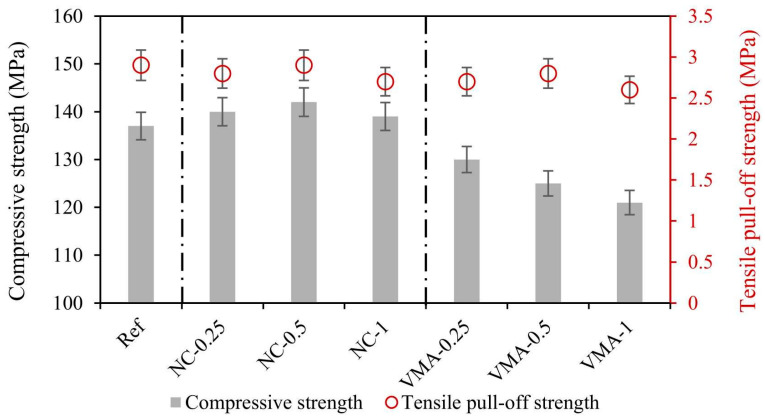
Coupled effect of NC and PCE or cellulose-based VMA and PCE on compressive strength and tensile pull-off strength, reprinted from [142], Copyright (2024), with permission from CC BY 4.0 license.

PAM-based VMAs provide a typical example of this performance differentiation. Yuan et al. found that although anionic, cationic, and nonionic PAMs reduced the compressive strength of cementitious matrix at most curing ages, anionic PAM increased the flexural strength by approximately 40% compared with the blank group at 3d [35]. Yao et al. also reported that anionic PAM can form microgels and complex with Ca^2+^, thereby enhancing interparticle attraction and improving the early flexural performance [34]. Meanwhile, the PAM-induced regulation of CH morphology and the interfacial transition zone may improve the local interfacial state and modify crack propagation paths [83]. Teng et al. incorporated nano-clay and cellulose ether-based VMA into UHPC with PCE, respectively, and found that VMA addition significantly reduced the compressive performance, while exerting only a limited effect on tensile strength, as shown in Figure 9 [142]. These findings indicate that the effect of VMAs on the flexural performance does not always vary in parallel with the compressive strength, but depends on whether the interfacial structure and the crack propagation mechanism are improved.

From the perspective of organic–inorganic composite structures, water-soluble VMAs may interact with Ca^2+^ and hydration products during hydration, forming film-like, fibrous, or network-like polymer structures on the surfaces of AFt, AFm, CH, and C-S-H, or within pore regions [106,107,108]. These polymer structures can strengthen the connection between hydration products and particles, thereby improving internal cohesion and the interfacial bonding [88,107,108]. Compared with inorganic hydration products, polymer films generally have higher deformability and a lower elastic modulus, allowing them to stretch, bend, or bridge under loading and thus contribute to energy absorption and delayed crack propagation [109]. After crack formation, the polymer films or network structures may further act as bridges, suppressing crack penetration and improving toughness and crack resistance [110].

### 5.3. Mechanisms Governing Mechanical Performance

Based on existing studies, the effects of VMAs on the mechanical performance of hardened cementitious materials are governed by the combined actions of hydration retardation, air entrainment and pore structure coarsening, water retention-induced hydration promotion, polymer film formation, and interfacial bridging. These mechanisms often occur simultaneously, but their relative contributions differ among mechanical indicators. The compressive strength is mainly controlled by the hydration degree, porosity, capillary pore connectivity, and the integrity of the load-bearing skeleton. Therefore, hydration retardation, air entrainment, and pore structure coarsening usually have more direct negative effects on compressive strength [101,102,103,104,105]. In contrast, the flexural strength, tensile behavior, and toughness are more sensitive to interfacial bonding, crack propagation paths, and energy dissipation. Polymer films, particle-bridging structures, and organic–inorganic interfacial interactions may therefore provide more pronounced benefits under tensile or flexural loading [88,106,107,108,109,110].

Therefore, VMAs should not be regarded as simply beneficial or detrimental to the hardened mechanical properties. Instead, they shift the dominant controlling mechanisms under different loading modes. For structural concrete, where the compressive load-bearing capacity is the primary requirement, the VMA dosage should be carefully controlled, with particular attention to hydration retardation, air entrainment, and pore-structure coarsening. For repair, grouting, rendering, and 3D-printed materials, where crack resistance, adhesion, interlayer bonding, and deformation compatibility are often more important, the interfacial regulation, flexible bridging, and toughening effects of VMAs may be more valuable. Accordingly, the mechanical performance should be evaluated according to the target application scenario rather than solely by compressive strength.

## 6. Effects of VMAs on Long-Term Durability of Cementitious Materials

### 6.1. Ions Transport and Leaching Resistance

The influence of VMAs on transport properties of cementitious materials is first reflected in their regulation of ions and moisture migration. Some VMAs can enhance the resistance of cementitious materials to aggressive ions ingress by increasing the pore solution viscosity and reducing the ions diffusion rate [115,116,117,118,119]. This indicates that, beyond conventional solid phase densification, pore solution viscosity regulation may provide an additional pathway for controlling the transport behavior. Bentz et al. introduced nanoscale VMAs into mortars through direct addition, absorption by porous lightweight aggregates, and surface impregnation. They found that these VMAs reduced the diffusion coefficient of ions in solution and significantly improved the resistance of the materials to chloride ingress and sulfate attack [115,116,117]. Khayat et al. also found that both Welan gum and HPMC reduced the chloride diffusion coefficient of concretes with different water-to-cement ratios, with a more pronounced improvement at lower water-to-cement ratios; among them, HPMC exhibited a stronger reducing effect than Welan gum [119]. Singh et al. further reported that HEC contributed to improving the resistance of cementitious materials to seawater attack [118].

**Figure 10:**
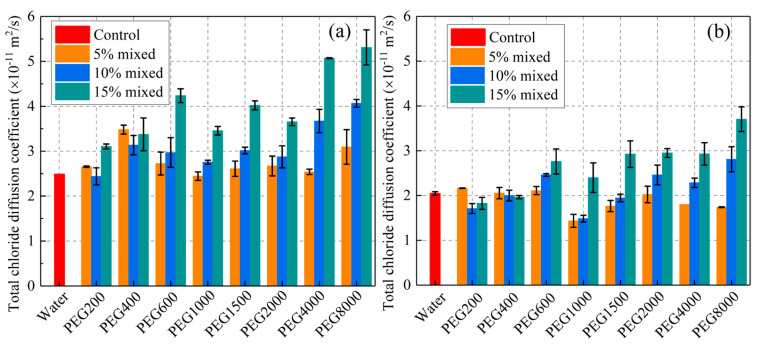
Effects of PEG molecular mass and dosage on the chloride diffusion coefficient of mortar under direct addition at different exposure ages: (**a**) 7 d; (**b**) 28 d, reprinted from [102], Copyright (2021), with permission from Elsevier.

However, the addition of VMAs does not invariably enhance the resistance to the ingress of deleterious media. When VMAs increase porosity, raise the proportion of capillary pores, or enhance pore connectivity, their adverse effects may offset or even exceed the benefits of liquid-phase viscosity-enhancing retardation. Shadkam et al. found that the incorporation of a polyvinyl-based VMA into self-leveling concrete increased the chloride diffusion coefficient, mainly because the VMA increased porosity and made the migration of aggressive media easier [120]. Zhao et al. directly added PEG into the mortar and found that, after 28 d of curing, the chloride diffusion coefficients of PEG-added mortars were higher than those of the control group after 7 d of chloride exposure. However, after 28 d of chloride exposure, mortars containing PEG with an appropriate molecular mass and dosage exhibited lower chloride diffusion coefficients than the control group, as shown in Figure 10. This behavior may be attributed to the influence of PEG addition on the chloride adsorption equilibrium within the matrix [102]. Therefore, the effect of VMAs on transport properties depends on the competition between beneficial effects, such as pore solution diffusion retardation, and adverse effects, such as pore structure coarsening, increased pore connectivity, and weakened ion binding [115,116,117,118,119,120].

Recent studies on different types of VMAs have further revealed this competitive relationship. PAM exerts a dual effect on chloride transport. On the one hand, by reducing the alkalinity of the pore solution, PAM enhances the chloride physical binding capacity of C-S-H; on the other hand, it reduces the chemical binding capacity by suppressing the formation of aluminate phases. Overall, the enhancement in the physical binding capacity exceeds the reduction in the chemical binding capacity, leading PAM-modified cement pastes to exhibit higher total chloride binding capacity and lower apparent chloride diffusion coefficients [128]. In contrast, CE-based VMAs may improve chloride binding capacity by reducing the alkalinity, but this benefit can be offset by an increased porosity, larger capillary pores, and a lower pore tortuosity. As a result, they generally increase the apparent chloride diffusion coefficient and weaken the resistance of cement pastes to chloride ingress. Under comparable conditions, the adverse effect of HPMC is usually stronger than that of HEC on chloride transport [129].

**Figure 11:**
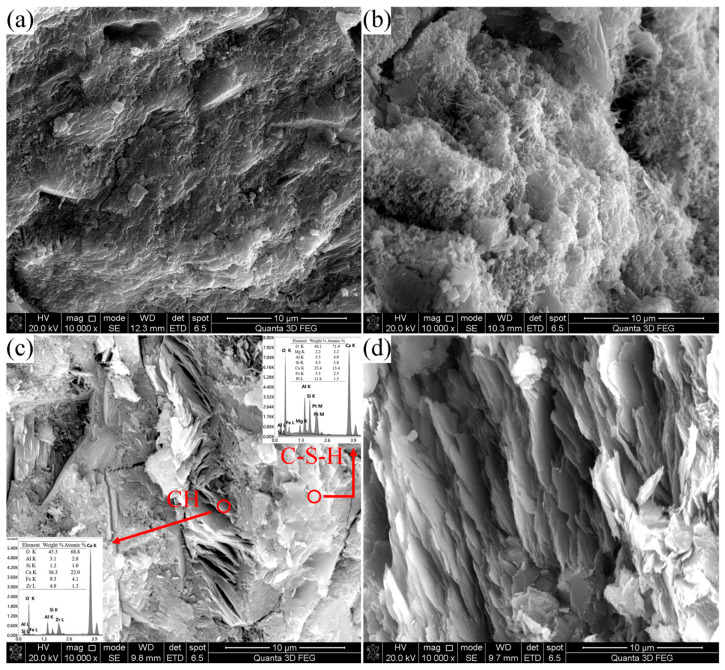
Morphological evolution of hydration products with PEG and DEIPA addition: (**a**) control; (**b**) 5% PEG; (**c**) 5% PEG and 0.03% DEIPA; (**d**) 5% PEG and 0.06% DEIPA, reprinted from [138], Copyright (2022), with permission from Elsevier.

CE-based VMAs may also weaken the resistance of cementitious materials to calcium leaching. Existing studies have shown that both HPMC and HEC reduce the content of CH and C-S-H due to hydration retardation, while also inducing more capillary pores and large pores. These changes increase the calcium leaching depth and reduce the pH of the materials, with HEC generally showing a stronger adverse effect than HPMC. Notably, the study further pointed out that calcium leaching deterioration is mainly governed by the content of CH and C-S-H rather than simply by the initial pore structure [130]. The combined incorporation of PEG and diethanol-isopropanolamine (DEIPA) into cement also increased the leaching susceptibility of the matrix. This may be attributed to pore structure coarsening, an increased Ca/Si ratio of C-S-H, and the plate-like morphology of the hydration products [138]. The evolution of the hydration product morphology with DEIPA dosage is shown in Figure 11.

In recent years, pore solution viscosity enhancement has attracted increasing attention as a durability regulation strategy. Related studies have proposed that pore solution viscosity enhancement can be achieved by replacing part of the mixing water, on an equal-mass basis, with an appropriate amount of a nonionic VMA, thereby slowing chloride ingress without significantly altering the porosity or mechanical performance. Among the investigated admixtures, PEG with a molecular mass of 1000 reduced the chloride diffusion coefficient by approximately 28% at 28d and also exhibited better resistance to calcium leaching [131]. This suggests that pore solution regulation may provide an alternative durability design strategy distinct from conventional densification-based approaches. Further studies have incorporated the pore solution viscosity-enhancing effect into the quantitative prediction of chloride transport, indicating that the chloride diffusion coefficient is controlled not only by the molecular shape factor of the VMA and its effective volume fraction in the pore solution, but also by VMA-induced pore structure coarsening and leaching resistance [132]. In addition, PEG may influence chloride binding behavior in mortars by modifying the C-S-H morphology and the AFm content [139]. These studies show that the regulation of transport behavior by VMAs is gradually shifting from empirical descriptions of durability phenomena toward quantitative correlations among pore solution microviscosity, ion diffusion, and pore structure evolution.

### 6.2. Carbonation Behavior

The influence of VMAs on the carbonation behavior is relatively complex and is mainly related to their effects on the CH content, the CH crystal size, the pore structure, and the deposition pattern of carbonation products [111,112,113,114]. The carbonation behavior should not be evaluated solely using the carbonation rate; instead, attention should also be paid to the microstructural reconstruction occurring during and after carbonation.

PAM-modified cement paste provides a typical example. Zhi et al. found that the carbonation rate of the cement paste increased with an increasing PAM content, whereas the carbonated samples became denser [111]. This was attributed to the reduction in the CH crystal size caused by PAM and the formation of finer and less crystalline calcium carbonate, which enabled the carbonation products to fill the pores more effectively. This indicates that the carbonation rate and the post-carbonation compactness should be distinguished: a higher carbonation rate does not necessarily correspond to a looser carbonated matrix.

Studies on CEs have reported markedly different results. Metalssi et al. suggested that variations in the CE dosage did not significantly affect the time required for complete carbonation of the specimens, but higher dosages slowed the carbonation kinetics and resulted in denser carbonated samples [112]. However, recent studies on CEs have shown that CEs may reduce the CH content in the cement paste and inhibit CH crystal growth, leading to smaller crystals with eroded edges [133]. At the same time, the air-entraining effect of CEs increases the porosity and coarsens the pore structure, thereby facilitating CO_2_ diffusion within the matrix and accelerating carbonation. This adverse effect becomes more pronounced with an increasing CE dosage, and HPMC generally shows higher carbonation sensitivity than HEC [133].

Other VMAs also exhibit different carbonation responses. Silva et al. found that incorporating modified potato starch into the lime mortar increased the porosity and coarsened the pore size distribution, thereby accelerating carbonation [113]. In contrast, Durán-Herrera et al., using a commercial low-molecular-mass shrinkage-reducing admixture as a VMA, reported that the VMA-containing concrete could still exhibit a relatively high carbonation resistance even at a lower strength level [114]. Therefore, the VMA-induced carbonation behavior should be understood from two coupled perspectives: the modification of CO_2_ diffusion and carbonation reaction conditions through changes in the CH content, the CH crystal size, and the pore structure; and the subsequent pore filling or microstructural reconstruction caused by carbonation products.

### 6.3. Shrinkage and Volumetric Stability

**Figure 12:**
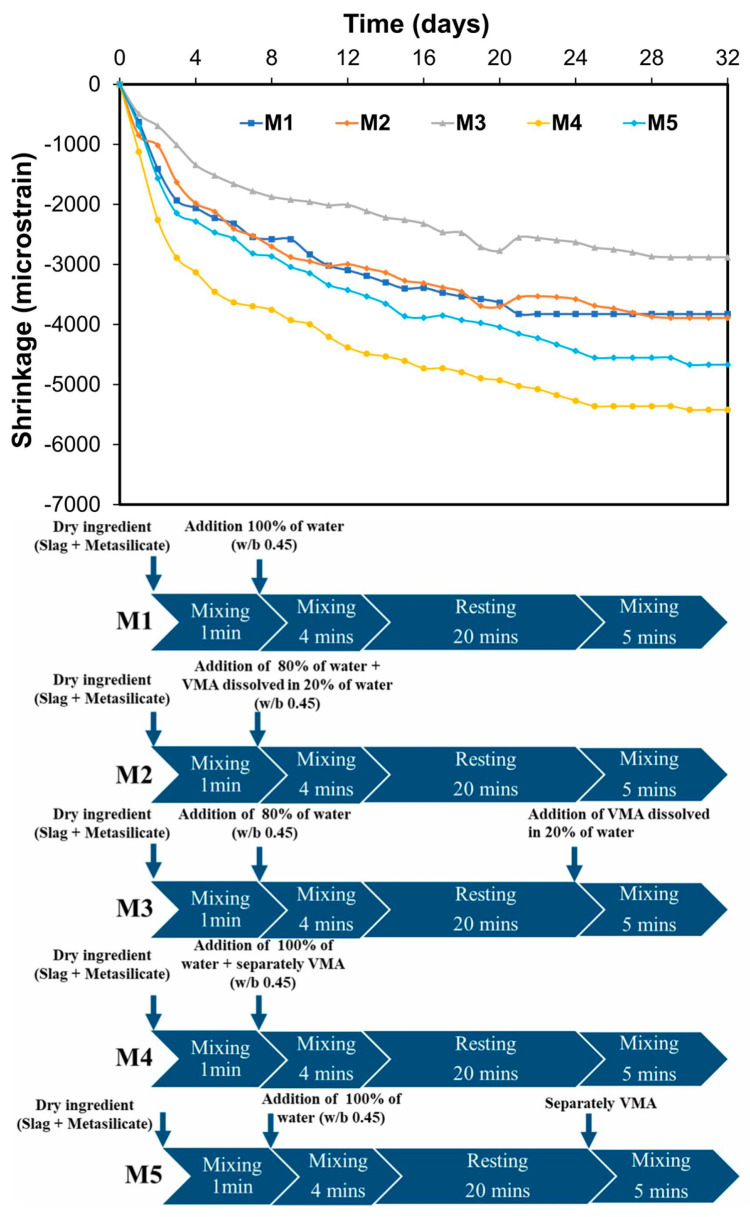
Effect of etherified starch addition method and timing on the drying shrinkage of alkali-activated slag systems, reprinted from [47], Copyright (2025), with permission from CC BY 4.0 license.

The influence of VMAs on shrinkage behavior also shows a clear difference. Many high-molecular-mass VMAs have been reported to increase drying shrinkage, whereas some low-molecular-mass VMAs may reduce shrinkage by lowering the surface tension and regulating the pore structure [35,104,115,121,122,123,124]. Yuan et al. found that different ionic PAMs all increased the drying shrinkage of the cement specimens, with the anionic PAM showing the most pronounced effect and the nonionic PAM showing a relatively weaker effect [35]. This was mainly attributed to the different effects of PAMs on internal moisture conditions and pore size distribution. Similarly, Wang et al. and Izaguirre et al. found that HEMC and Guar gum derivatives significantly increased the drying shrinkage in cement mortar and lime-based mortar, respectively, by increasing the proportion of capillary pores and the total porosity [121,122]. Even after controlling the air content, the adverse effect of HEMC on the drying shrinkage remained, indicating that its influence does not arise solely from air entrainment or total porosity, but is closely related to pore structure evolution [104]. But Ali et al. showed that the drying shrinkage of alkali-activated slag systems could be reduced by optimizing the incorporation method and timing of etherified starch [47], as shown in Figure 12.

In contrast, low-molecular-mass polyether-based VMAs exhibit shrinkage-reducing advantages. Bentz et al. found that low-molecular-mass VMAs could reduce the shrinkage of mortars containing supplementary cementitious materials through different addition methods [115]. In alkali-activated slag systems, low-molecular-mass polyethers such as PEG and polypropylene glycol (PPG) showed more pronounced advantages in reducing the drying shrinkage [123,124]. Bílek et al. reported that PEG significantly reduced the drying shrinkage of the alkali-activated slag mortar by increasing porosity, lowering the pore solution surface tension, and regulating the proportion of mesopores, with the effect jointly controlled by the molecular mass and dosage [123]. Ye et al. found that PPG could also suppress the shrinkage by reducing the pore solution surface tension and coarsening the pore structure, with the lower-molecular-mass PPG showing a stronger effect [124]. Although alkali-activated slag differs from OPC in the pore solution chemistry and reaction products, these studies highlight the importance of surface tension regulation in shrinkage control.

These findings indicate that pore coarsening does not necessarily increase the shrinkage when surface tension reduction dominates. When low-molecular-mass polyether-based VMAs significantly reduce the surface tension of the pore solution, the capillary pressure decreases accordingly; thus, the shrinkage may still be reduced even if the pore structure is coarsened. Therefore, the effect of VMAs on the volumetric stability should be understood based on the coupled relationships among the pore size distribution, the surface tension, and the internal moisture state, rather than being judged simply from changes in porosity.

### 6.4. Freeze–Thaw Resistance

The influence of VMAs on the freeze–thaw resistance is mainly associated with their regulation of porosity, the pore size distribution, and the air-void system [2,122,125,126,127]. Unlike chloride transport or carbonation, the freeze–thaw resistance depends not only on the total porosity, and the pore size distribution, but more strongly on the air-void spacing factor, and the ability to release freezing pressure.

Some studies have shown that VMAs can improve the freeze–thaw resistance by optimizing the pore structure or the air-void system. Izaguirre et al. found that HEMC and Guar gum derivatives improved the freeze–thaw resistance of the mortar by increasing the porosity and concentrating the pore size distribution [122]. Yu et al. improved the freeze–thaw resistance of concrete by synergistically regulating the pore structure using a defoamer and a VMA [125]. Łaźniewska-Piekarczyk also observed that the incorporation of MC into self-compacting concrete improved freeze–thaw resistance due to an increased porosity [126]. These results indicate that an appropriate pore- and air-void structure can provide buffer space for the expansion of freezing water, thereby improving freeze–thaw stability.

However, not all VMAs improve the freeze–thaw resistance. Khayat, citing the work of Yamato et al., noted that concrete mixtures containing cellulose-based or PAM-based VMAs may exhibit poorer freeze–thaw resistance than concrete without VMAs, with the adverse effect being more pronounced in the cellulose-based VMA systems [2,127]. This was partly attributed to the increased maximum air-void spacing following VMA incorporation. A larger spacing factor means that the freezing water has fewer nearby pressure-relief voids, which can intensify the hydraulic and crystallization pressures. This indicates that the effect of VMAs on the freeze–thaw resistance is not governed by porosity alone, but is closely related to the air-void system parameters.

In 3D-printed cementitious materials, the influence of VMAs on the air-void system is further coupled with the printing process. A recent study has shown that HPMC is more effective in stabilizing small bubbles before printing. Meanwhile, the printing process itself can elongate the bubbles along the printing direction and induce an oriented bubble distribution [143]. Therefore, for 3D-printed concrete intended for cold regions or freeze–thaw environments, the VMA selection should not be based solely on rheological regulation; its influence on the air-void stability and the evolution of the final air-void structure should also be considered.

Overall, the durability-related effects of VMAs have been experimentally investigated in terms of ion transport, carbonation, shrinkage, and freeze–thaw resistance. Among these aspects, ion transport and carbonation have been relatively more systematically studied, with evidence from diffusion tests, ions binding measurements, pore solution viscosity regulation, pore structure characterization, carbonation-product deposition and calcium leaching tests. Shrinkage and freeze–thaw resistance have also been experimentally examined, but the available studies are more system-specific and the mechanisms are more complex. Therefore, future studies should further separate the coupled factors and establish clearer mechanism–performance relationships under different binder systems and exposure conditions.

## 7. Structure–Mechanism–Performance Relationships and Mixture Design Principles

### 7.1. Structure–Mechanism–Performance Relationship

Overall, the role of water-soluble VMAs in cementitious materials can be understood as a continuous pathway linking molecular structure, action mechanism, microstructural evolution, and macroscopic performance. The VMA molecular parameters, including the molecular mass, functional groups, substituent type and degree, and charge characteristics, strongly govern their conformation in the pore solution, interactions with water molecules, adsorption tendency on particle and hydration-product surfaces, and response to ions such as Ca^2+^ [17,21,22,23,24,25,27,36,37,38,39]. Among these parameters, the molecular mass and chain conformation mainly affect the liquid-phase viscosity, chain entanglement, and network formation; the functional groups and substituent composition mainly influence adsorption, nucleation, and the growth of hydration products; and the charge characteristics regulate the interactions of VMAs with ions, particle surfaces, and superplasticizers [1,2,3,4,12,13,14,15,16,17,18,31,32,33,34,35,44,45,82,83]. Therefore, the VMA performance cannot be inferred simply from material category or viscosity-enhancing ability. Instead, it should be analyzed according to the dominant mechanisms activated and the boundary conditions under which these mechanisms operate. To further clarify this structure–mechanism–performance framework, representative water-soluble VMAs are compared in terms of their dominant mechanisms, typical performance implications, and key compatibility or design considerations in Table 1. The table highlights that the same VMA type may produce beneficial or adverse effects depending on whether the dominant mechanism is liquid-phase thickening, adsorption-controlled flocculation, hydration retardation, pore-structure modification, or interfacial bridging.

VMAs effects extend beyond fresh state regulation and propagate into the hardened properties through hydration kinetics, hydration product formation, pore structure evolution, and organic–inorganic interfacial development [36,37,38,39,41,42,80,81,83,84,85,86,87,88,89,90,91,92,93,95,96,97,98]. Many water-soluble VMAs tend to prolong the induction period, delay the main heat flow peak, or inhibit early nucleation processes [36,37,38,39,41,42,80,81,83,84,85,86,87,88,89,90,91,92,93,95,96,97,98], thereby affecting the precipitation mode, crystal morphology, and distribution of C-S-H, CH, and aluminate hydration products [41,42,83,84,85,86,87,88,95,96,97,98]. These effects may promote later hydration at low dosages through water retention, but may also lead to a looser microstructure at high dosages due to hydration retardation and altered product packing [84,85,86,87,88]. Meanwhile, the formation of films, fibrous structures, or network-like structures of polymer indicates that VMAs may also participate in the microstructural construction as an organic phase, modifying the connection between the hydration products and the crack propagation path [88,106,107,108,109,110].

Because VMA-induced microstructural evolution is dual and multiscale, its effects on the macroscopic properties are highly differentiated. The compressive strength is primarily governed by porosity, capillary pore connectivity, and load-bearing skeleton integrity. The flexural strength, tensile behavior, and toughness are more sensitive to interfacial bonding, bridging structures, and crack propagation paths. The transport and durability performance are controlled by the competition among pore solution diffusion retardation, ion binding, hydration product composition, and pore structure reconstruction [34,35,83,88,101,102,103,104,105,106,107,108,109,110,111,112,113,114,115,116,117,118,119,120,121,122,123,124,125,126,127]. Therefore, the macroscopic effect of a VMA should be evaluated according to the controlling mechanisms of the target property, rather than through a single indicator such as viscosity, flowability, or compressive strength.

Taken together, these findings indicate that the VMA performance should be interpreted through a mechanism-based framework rather than through a direct comparison of individual studies. The same VMA may produce different or even opposite performance responses when the dominant mechanism shifts from liquid-phase thickening to adsorption-controlled flocculation, from water-retention-induced hydration promotion to retardation-induced pore coarsening, or from pore-solution diffusion retardation to connectivity-controlled transport. Therefore, the key issue is not whether a given VMA is intrinsically beneficial or detrimental, but under which binder chemistry, dosage range, admixture combination, and service condition a specific mechanism becomes dominant.

### 7.2. Origin of Inconsistent Findings

The inconsistent or even contradictory conclusions reported for the same type of VMA mainly arise from differences in the system boundary conditions. First, binder chemistry plays a fundamental role. OPC, LC^3^, sulfoaluminate cement, alkali-activated materials, and lime-based materials differ markedly in their mineral composition, particle surface properties, pore solution chemistry, and hydration product assemblages. These differences directly affect the VMA dissolution, molecular conformation, adsorption, complexation, water retention, and hydration retardation [36,37,38,39,41,42,80,81,84,85,86,87,88,89,90,91,92,93,95,96,97,98].

Second, the water-to-binder ratio, the solid volume fraction, and the particle gradation also change the dominant rheological mechanisms. In systems with low solid volume fractions, changes in the liquid-phase viscosity may dominate; whereas in systems with high solid volume fractions, such as UHPC or 3D-printing materials, the interparticle distance, the flocculated networks, and the structural build-up rate are often more critical [12,13,14,15,16,17,18,21,22,23,24,25,27,44,45,48,49,50,59,60,61].

Third, the admixture compatibility strongly affects the VMA performance. Competitive adsorption or synergistic interactions between VMAs and superplasticizers can alter the dispersion efficiency, the flowability, the structural build-up, the air entrainment, and the hydration kinetics [12,13,14,15,16,17,18,44,45,46,67,68]. Therefore, conclusions obtained from single-admixture systems cannot be directly transferred to mixtures containing PCEs, defoamers, supplementary cementitious materials, or other functional components.

Fourth, the dosage range and the curing age influence whether beneficial or adverse mechanisms become dominant. Low VMA dosages may improve water retention, stability, and later hydration, whereas excessive dosages may cause over-thickening, flowability loss, hydration retardation, air entrainment, or pore-structure coarsening [34,35,61,80,101,102,103,104,105]. Similarly, early-age results may mainly reflect retardation or structural build-up, while later-age properties are more strongly affected by continued hydration, pore-structure evolution, and polymer–hydration product interactions.

Finally, the testing method and evaluation criteria also contribute to inconsistent conclusions. For example, the reported yield stress may correspond to the static yield stress, the dynamic yield stress, or the protocol-dependent apparent yield stress, depending on the rheological test procedure. Similarly, durability indicators may differ according to exposure regime, conditioning method, and testing age. Therefore, the VMA effects should be evaluated with explicit consideration of binder chemistry, water-to-binder ratio, admixture combination, dosage range, curing age, and test method, rather than through generalized judgments detached from their boundary conditions.

### 7.3. Mixture Design Principles for Different Applications

From an engineering application perspective, the purpose of using VMAs is not simply to increase the viscosity, but to achieve balanced performance under specific construction and service requirements. The mixture design should start from the target performance deficit rather than from VMA dosage. The selected VMA should activate the desired dominant mechanism while minimizing side effects such as excessive viscosity, hydration retardation, air entrainment, pore-structure coarsening, or delayed strength development.

Practically, VMA selection should follow a target-oriented logic. When the main problem is insufficient stability in highly flowable mixtures, VMAs with strong water retention, liquid-phase viscosity enhancing, or moderate adsorption capacity are preferred, but their compatibility with superplasticizers must be verified. When the target is 3D printability, the selected VMA should increase the static yield stress and structural build-up after deposition without excessively increasing the dynamic yield stress and extrusion pressure. When the target is grouting or repair, water retention, bleeding resistance, injectability, adhesion, and operation-dependent open time should be considered simultaneously. When the target is durability improvement, VMAs should be selected according to the intended mechanism, such as pore-solution viscosity regulation for ion transport control, surface tension reduction for shrinkage mitigation, or air-void stabilization for freeze–thaw resistance. Therefore, the same VMA may be suitable for one performance target but unsuitable for another if the dominant mechanism conflicts with the required construction or service performance.

For self-compacting concrete and other highly flowable materials, the design focus lies in coordinating the flowability and stability [6,7]. Slump flow, bleeding, segregation, flow retention, and aggregate suspension stability should be evaluated simultaneously [12,13,14,15,16,17,18,44,45]. For 3D-printed materials, the VMA design should focus on balancing pumpability, extrudability, static structural recovery, buildability, and the printable window [51,52,53,54,55,56,57,58,76]. For grouting materials, repair materials, and similar systems, water retention, bleeding resistance, construction adhesion, and operation-dependent open time are the main requirements. In some grouting operations, moderate hydration retardation can be beneficial for maintaining injectability and preventing premature stiffening, whereas excessive retardation should still be avoided when early strength development or rapid service recovery is required [5,20,36,37,38,39,41,42,101,102,103,104,105]. For durability-oriented systems, pore-solution diffusion retardation, pore-structure evolution, shrinkage behavior, and air-void system should be comprehensively assessed [111,112,113,114,115,116,117,118,119,120,121,122,123,124,125,126,127,128,129,130,131,132,143].

In addition to the VMA type and dosage, the addition sequence during mixing is also an important factor affecting the mixture performance. Pre-dissolving VMAs in mixing water, adding them together with superplasticizers, or introducing them after initial dispersion may lead to different degrees of polymer dissolution, swelling, adsorption, chain entanglement, and competitive interaction with PCEs. Therefore, the same VMA may produce different effects on flowability, viscosity, structural build-up, air entrainment, and hydration depending on when and how it is incorporated. This issue is particularly important in systems containing both VMAs and superplasticizers, as well as in 3D-printed materials where secondary addition or secondary mixing can be used to improve the static yield stress while limiting the increase in the dynamic yield stress [47,51]. Therefore, the VMA addition sequence and mixing shear history should be considered as part of mixture design, rather than being treated as only experimental details.

Overall, the VMA design is a multi-objective, multiscale mixture optimization problem rather than a simple dosage adjustment. An effective design strategy should link molecular structure, action mechanism, microstructural evolution, and target performance, while also considering the specific binder system, admixture combination, construction process, and service environment.

## 8. Research Gaps and Future Perspectives

Overall, existing studies have clarified the effects of VMAs on fresh-state rheology and construction stability. However, a systematic understanding remains limited regarding how molecular structural parameters quantitatively govern their mechanisms of action, how VMAs perform in low-carbon and non-OPC binders, and how they affect long-term durability under service-relevant conditions. Future research should focus on quantitative structure–mechanism–performance relationships, binder-specific applicability, long-term durability assessment and service-life prediction, and green functional design, thereby promoting the development of VMAs from empirical use toward predictive design.

### 8.1. Quantitative Structure–Mechanism–Performance Relationships

Existing studies have shown that the VMA molecular parameters, including molecular mass, functional groups, substituent type, degree of substitution, and charge characteristics, are key factors controlling their effects in cementitious materials [17,21,22,23,24,25,27,36,37,38,39]. However, quantitative relationships between these molecular parameters and rheological regulation, hydration retardation, pore structure evolution, mechanical performance, and durability responses remain unclear. Current studies can explain why different VMAs behave differently, but they cannot yet predict how changes in specific molecular parameters translate into rheological, hydration, microstructural, mechanical, or durability responses.

This limitation mainly arises from two aspects. First, most studies focus on commercial admixtures or individual polymer types, while systematic investigations controlling single structural variables, such as molecular mass, degree of substitution, functional group density, or charge density, remain insufficient. Second, differences in cement type, water-to-binder ratio, supplementary cementitious materials, superplasticizer type, and dosage range reduce the comparability of reported results. Future studies should establish molecular-structure-centered databases and predictive models that correlate molecular descriptors with yield stress, plastic viscosity, thixotropy, hydration heat, pore structure, strength, and durability parameters. Only with such advances can the VMA selection shift from empirical screening to demand-oriented molecular design.

### 8.2. Applicability in Low-Carbon and Non-OPC Binders

Current research on VMAs is still mainly based on OPC systems, while their applicability in alkali-activated materials, sulfoaluminate cement, magnesium phosphate cement, and other low-carbon or non-OPC binders remains insufficiently understood [105,123,124]. Different binder systems vary significantly in mineral composition, particle surface properties, pore solution chemistry, and hydration products, which directly affect the VMA adsorption, complexation, and retardation behavior. Therefore, the empirical rules derived from OPC systems cannot be directly extrapolated to emerging binders.

LC^3^ is a representative low-carbon binder in which the VMA behavior may differ significantly from that in OPC. Calcined clay has a high specific surface area, a layered and rough particle morphology, a strong flocculation tendency, and a high admixture adsorption capacity, which generally increase yield stress, plastic viscosity, and superplasticizer demand [134]. Moreover, the additional aluminate phases and Ca^2+^ sources in LC^3^ systems may modify VMA-induced retardation and alter the balance between rheological regulation and early hydration [94]. VMAs should not be used merely as stability-enhancing agents, but should be designed in coordination with superplasticizers, fine particles, particle packing, and hydration reactions.

Alkali-activated binders represent another important class of low-carbon systems with distinct pore solution chemistry. Compared with OPC, alkali-activated slag or fly ash systems usually contain higher alkalinity, higher ionic strength, and different dominant reaction products, such as C-(A)-S-H or N-A-S-H gels. These characteristics may alter the stability, dissolution, adsorption, and viscosity-enhancing efficiency of water-soluble polymers. Recent studies have shown that the addition method of VMAs can significantly affect the rheological behavior of alkali-activated slag systems [47]. Meanwhile, low-molecular-mass polyethers such as PEG and PPG can reduce drying shrinkage in alkali-activated slag mortars by regulating pore solution surface tension and pore structure, but these effects are strongly dependent on molecular mass, dosage, activator chemistry, and binder composition [123,124]. Therefore, the VMA functions in alkali-activated systems should be evaluated together with activator type, alkali concentration, Ca availability, reaction-product assemblage, and pore solution evolution.

Other non-OPC binders also require binder-specific VMA design. In sulfoaluminate cement systems, rapid ettringite formation and sulfate–aluminate reactions may change the sensitivity of hydration, setting, and strength development [105]. In binders with markedly different pore chemistries, the ion-responsive behavior, alkaline stability, and adsorption mode of VMAs may differ from those in OPC. Future studies should, therefore, establish binder-specific VMA design principles based on pore solution chemistry, particle surface properties, reaction products, admixture compatibility, addition sequence, and service requirements, rather than relying on OPC-based experience alone.

### 8.3. Long-Term Durability and Service-Life Prediction

Compared with fresh-state properties and early-age mechanical performance, research on the long-term durability effects of VMAs remains relatively limited [111,112,113,114,115,116,117,118,119,120,121,122,123,124,125,126,127]. Existing studies show that VMAs may improve the resistance to aggressive media by increasing the pore solution viscosity and reducing te ion migration, but they may also weaken certain durability indicators through air entrainment, pore structure coarsening, or increased shrinkage. Overall, most available studies still focus on individual durability indicators, while systematic investigations coupling transport behavior, pore structure evolution, volumetric stability, chemical degradation, and environmental response remain insufficient.

Future research should evaluate VMA-modified cementitious materials under service-life-relevant exposure conditions. Longer-term tests under realistic or coupled environments are needed to avoid generalized conclusions based only on short-term accelerated experiments. Meanwhile, durability and service-life prediction models should incorporate multiple coupled mechanisms, including pore solution viscosity, ion binding, pore connectivity, hydration products content, shrinkage evolution, and air-void structure. Under coupled exposures involving chloride ingress, carbonation, freeze–thaw cycling, calcium leaching, or salt freezing, future studies should clarify whether VMAs improve durability through pore solution diffusion retardation or introduce long-term risks through pore-structure coarsening and hydration-product modification.

### 8.4. Green, Functional, and Digital Design of VMAs

In the context of low-carbon construction materials and advanced construction technologies, the future development of VMAs should move beyond the conventional liquid-phase viscosity enhancement and stabilization functions toward a green, functional, and scenario-oriented design. On the one hand, greater attention should be paid to renewable, low-impact, biodegradable, or more sustainably produced VMAs, such as low-environmental-burden natural polysaccharides and their derivatives [81,144], as well as plant-based biopolymers [19]. On the other hand, function-oriented VMAs should be developed for specific engineering needs, including highly thixotropic VMAs with rapid structural recovery for 3D printing, liquid-phase-retarding VMAs for durability enhancement, surface-tension-regulating polyether VMAs for low-shrinkage materials, and air-void-stabilizing VMAs for freeze–thaw environments [51,52,53,54,55,56,57,58,115,116,117,118,119,120,121,122,123,124].

Extreme environments and special engineering scenarios also impose new functional requirements on VMAs. For high-temperature deep-well applications, organic–inorganic hybrid viscosity-enhancing and slurry-stabilizing admixtures have shown potential for improving thermal stability, suspension stability, and fluid-loss control [145]. For digital construction, VMAs should be co-designed with printing paths, nozzle shear, interlayer deposition, and air-void evolution. Future work may further integrate molecular simulation, multiscale in situ characterization, and machine-learning-assisted modeling to link molecular descriptors, pore solution chemistry, rheological response, hydration kinetics, microstructural evolution, and long-term performance. This would help establish a data-driven VMA development framework connecting molecular design with the macroscopic performance prediction.

## 9. Conclusions

This review systematically analyzed the effects and mechanisms of water-soluble VMAs in cementitious materials by linking molecular structure, action mechanisms, microstructural evolution, and macroscopic performance. The reviewed literature shows that the VMA performance cannot be predicted solely from admixture type or dosage. Instead, it is governed by molecular parameters, including molecular mass, functional groups, substituent type and degree of substitution, charge characteristics, and chain structure, together with binder composition, pore solution chemistry, admixture compatibility, mixing procedure, and service environment.

Several mechanisms can be considered relatively well established. In the fresh state, liquid-phase viscosity enhancement, water retention, polymer-chain entanglement, particle adsorption/bridging, and VMA–superplasticizer interactions control plastic viscosity, flowability, segregation resistance, structural build-up, and printability. In hydration and microstructure development, VMAs commonly modify hydration kinetics, nucleation, hydration product morphology, pore structure, and organic–inorganic interfacial formation. In hardened materials, hydration retardation, air entrainment, and pore structure coarsening often reduce compressive strength, whereas polymer-film formation, interfacial bridging, and crack-bridging effects may improve flexural behavior and toughness. For durability, pore solution viscosity regulation, ion binding, pore connectivity, CH/C–S–H content, surface-tension regulation, and air-void structure have been identified as key factors controlling ion transport, leaching, carbonation, shrinkage, and freeze–thaw resistance.

The most important unresolved issues are the quantitative prediction of VMA effects and their adaptability across binder systems. Future research should prioritize: establishing quantitative structure–mechanism–performance relationships; clarifying the VMA behavior in LC^3^, alkali-activated binders, sulfoaluminate cement, and other non-OPC systems with different pore chemistries; separating the effects of VMA molecular structure, dosage, addition sequence, and shear history; and evaluating long-term durability under coupled service-relevant environments. Addressing these issues will help shift water-soluble VMAs from empirically selected admixtures toward predictable, designable, and multifunctional performance-regulating components for conventional and emerging cementitious materials.

## Figures and Tables

**Table 1:** Comparative summary of representative water-soluble VMAs, dominant mechanisms, performance effects, and design considerations.

VMA Type	Representative Examples	Dominant Mechanisms	Typical Performance Effects	Compatibility/Design Notes
Polysaccharide-based VMAs and derivatives	Cellulose ethers, including MC, HEC, HEMC, and HPMC; microbial polysaccharides, such as Welan gum and Diutan gum; plant-derived derivatives, such as Guar gum, hydroxypropyl guar, and starch derivatives [18,20,24,27,28,29,30,36,37,38,39,46,57,64,80,81,122,144]	Liquid-phase viscosity enhancement, water retention, chain entanglement, adsorption or bridging, and substitution- or charge-dependent interactions with Ca^2+^ and hydration products [20,24,25,27,28,29,30,36,37,38,39,46,57,64,80,81]	Improve cohesion, plastic viscosity, water retention, bleeding resistance, segregation resistance, and shape retention; may enhance structural build-up, but excessive dosage may reduce flowability, retard hydration, increase porosity, or reduce compressive strength [28,30,48,57,59,61,64,67,101,103,104,105,113,119,122,129,130,133]	Molecular structure, substitution degree, charge characteristics, and superplasticizer compatibility are critical; competitive adsorption, abnormal thickening, gel formation, or increased superplasticizer demand may occur [12,13,17,29,44,45,46,64,67,68,81,144]
Polyacrylamide-based VMAs	Nonionic PAM, anionic PAM, cationic PAM [34,35,56,73,82]	Chain entanglement, particle bridging, charge-dependent adsorption, Ca^2+^ complexation, and flocculation regulation [34,35,56,73,82]	Regulate plastic viscosity, cohesion, yield stress, structural build-up, and workability, with the effects depending on molecular mass, charge characteristics, and dosage; may reduce compressive strength but improve early flexural performance or reduce chloride diffusion [34,35,73,83,128,136,137]	Charge density, molecular mass, and dosage should be controlled; excessive bridging or flocculation may cause flowability loss [34,35,56,58,73,82]
Other ionic or carboxylate-containing polymers	Sodium alginate, carboxymethyl chitosan, polyacrylic acid-based polymers [31,32,33]	Ion-responsive behavior, Ca^2+^ complexation, adsorption, ionic bridging, and possible local gelation [31,32,33]	Increase yield stress, viscosity, and structural build-up; may improve stability at suitable dosages [31,32,33]	Highly sensitive to Ca^2+^ concentration, pH, and superplasticizer type; competitive adsorption with PCEs should be considered [32,33]
Polyether-based water-soluble polymers	PEG, PPG and related low-molecular-mass polyethers [98,102,115,123,124,131,132,138,139]	Pore-solution viscosity regulation, water regulation, surface-tension reduction, and modification of C-S-H morphology or packing [98,102,123,124,131,132,139]	More relevant to transport and shrinkage control than conventional fresh-state stabilization; may reduce chloride diffusion or drying shrinkage when pore coarsening is controlled [102,115,123,124,131,132,139]	Effects depend strongly on molecular mass, dosage, and binder chemistry; especially important in durability-oriented design [102,123,124,131,132]
Film-forming water-soluble polymers	PVA, PVAA and related polymers [88,106,107,108,109,110]	Polymer-film formation, organic–inorganic interfacial bonding, crack bridging, and energy dissipation [88,106,107,108,109,110]	Improve cohesion, adhesion, flexural toughness, crack resistance, and interfacial bonding; compressive strength may decrease if porosity or air entrainment increases [88,106,107,108,109,110]	Particularly relevant to repair, rendering, grouting, and interfacial bonding applications; polymer stability and compatibility with hydration products should be considered [88,107,108,109,110]

## Data Availability

No new data were created or analyzed in this study. Data sharing is not applicable to this article.

## References

[1] Khayat K.H. (1995). Effect of antiwashout admixtures on fresh concrete properties. ACI Mater. J..

[2] Khayat K.H. (1998). Viscosity-enhancing admixtures for cement-based materials—An overview. Cem. Concr. Comp..

[3] Khayat K.H., Mikanovic N., Roussel N. (2012). 8—Viscosity-enhancing admixtures and the rheology of concrete. Understanding the Rheology of Concrete.

[4] Yammamuro H., Mizunuma T. (1997). Study of non-adsorptive viscosity agents applied to self-compacting concrete. Spec. Publ..

[5] Khayat K., Yahia A., Duffy P. (1999). High-performance cement grout for post-tensioning applications. Mater. J..

[6] Sari M., Prat E., Labastire J.-F. (1999). High strength self-compacting concrete original solutions associating organic and inorganic admixtures. Cem. Concr. Res..

[7] Rols S., Ambroise J., Pera J. (1999). Effects of different viscosity agents on the properties of self-leveling concrete. Cem. Concr. Res..

[8] Kamal M.M., Safan M.A., Bashandy A.A., Khalil A.M. (2018). Experimental investigation on the behavior of normal strength and high strength self-curing self-compacting concrete. J. Build. Eng..

[9] Sri Rama Chand M., Swamy Naga Ratna Giri P., Rathish Kumar P., Rajesh Kumar G., Raveena C. (2016). Effect of self curing chemicals in self compacting mortars. Constr. Build. Mater..

[10] Hou S., Duan Z., Xiao J., Ye J. (2021). A review of 3D printed concrete: Performance requirements, testing measurements and mix design. Constr. Build. Mater..

[11] Souza M.T., Ferreira I.M., Guzi de Moraes E., Senff L., Novaes de Oliveira A.P. (2020). 3D printed concrete for large-scale buildings: An overview of rheology, printing parameters, chemical admixtures, reinforcements, and economic and environmental prospects. J. Build. Eng..

[12] Kawakami M., Wada S., Suzukawa K. (1989). Effects of Chemical Admixtures onColloidal Underwater Concrete. Spec. Publ..

[13] Kawai T., Okada T. (1989). Effect of superplasticizer and viscosity-increasing admixture on properties of lightweight aggregate concrete. Spec. Publ..

[14] Wu Y., He T., Song X., Liang G. (2011). Effect of sodium gluconate on polynaphthalene sulfonate adsorption. Adv. Cem. Res..

[15] Tan H., Ma B., Li X., Jian S., Yang H. (2014). Effect of competitive adsorption between sodium tripolyphosphate and naphthalene superplasticizer on fluidity of cement paste. J. Wuhan Univ. Technol.-Mater. Sci. Ed..

[16] Plank J., Winter C. (2008). Competitive adsorption between superplasticizer and retarder molecules on mineral binder surface. Cem. Concr. Res..

[17] Ma B., Peng Y., Tan H., Jian S., Zhi Z., Guo Y., Qi H., Zhang T., He X. (2018). Effect of hydroxypropyl-methyl cellulose ether on rheology of cement paste plasticized by polycarboxylate superplasticizer. Constr. Build. Mater..

[18] Tan H., Zou F., Ma B., Guo Y., Li X., Mei J. (2017). Effect of competitive adsorption between sodium gluconate and polycarboxylate superplasticizer on rheology of cement paste. Constr. Build. Mater..

[19] Boutouam Y., Hayek M., Bouarab K., Yahia A. (2024). A Comprehensive Review of Plant-Based Biopolymers as Viscosity-Modifying Admixtures in Cement-Based Materials. Appl. Sci..

[20] Brumaud C., Baumann R., Schmitz M., Radler M., Roussel N. (2014). Cellulose ethers and yield stress of cement pastes. Cem. Concr. Res..

[21] Ferraris C.F., Obla K.H., Hill R. (2001). The influence of mineral admixtures on the rheology of cement paste and concrete. Cem. Concr. Res..

[22] Seabra M.P., Paiva H., Labrincha J.A., Ferreira V.M. (2009). Admixtures effect on fresh state properties of aerial lime based mortars. Constr. Build. Mater..

[23] Paiva H., Esteves L.P., Cachim P.B., Ferreira V.M. (2009). Rheology and hardened properties of single-coat render mortars with different types of water retaining agents. Constr. Build. Mater..

[24] Patural L., Marchal P., Govin A., Grosseau P., Ruot B., Devès O. (2011). Cellulose ethers influence on water retention and consistency in cement-based mortars. Cem. Concr. Res..

[25] Hot J., Bessaies-Bey H., Brumaud C., Duc M., Castella C., Roussel N. (2014). Adsorbing polymers and viscosity of cement pastes. Cem. Concr. Res..

[26] Kawai T. Non-dispersible underwater concrete using polymers. Proceedings of the 5th International Congress on Polymers.

[27] Khayat K., Yahia A. (1997). Effect of welan gum-high-range water reducer combinations on rheology of cement grout. Mater. J..

[28] Poinot T., Govin A., Grosseau P. (2014). Influence of hydroxypropylguars on rheological behavior of cement-based mortars. Cem. Concr. Res..

[29] Üzer E., Plank J. (2016). Impact of welan gum stabilizer on the dispersing performance of polycarboxylate superplasticizers. Cem. Concr. Res..

[30] Sonebi M. (2006). Rheological properties of grouts with viscosity modifying agents as diutan gum and welan gum incorporating pulverised fly ash. Cem. Concr. Res..

[31] Lasheras-Zubiate M., Navarro-Blasco I., Fernández J.M., Álvarez J.I. (2012). Effect of the addition of chitosan ethers on the fresh state properties of cement mortars. Cem. Concr. Comp..

[32] Ma B., Peng Y., Tan H., Lv Z., Deng X. (2018). Effect of Polyacrylic Acid on Rheology of Cement Paste Plasticized by Polycarboxylate Superplasticizer. Materials.

[33] Li M., Pan L., Li J., Xiong C. (2020). Competitive adsorption and interaction between sodium alginate and polycarboxylate superplasticizer in fresh cement paste. Colloids Surf. A Physicochem. Eng. Asp..

[34] Yao H., Fan M., Huang T., Yuan Q., Xie Z., Chen Z., Li Y., Wang J. (2021). Retardation and bridging effect of anionic polyacrylamide in cement paste and its relationship with early properties. Constr. Build. Mater..

[35] Yuan Q., Xie Z., Yao H., Huang T., Fan M. (2022). Hydration, mechanical properties, and microstructural characteristics of cement pastes with different ionic polyacrylamides: A comparative study. J. Build. Eng..

[36] Pourchez J., Grosseau P., Ruot B. (2009). Current understanding of cellulose ethers impact on the hydration of C3A and C3A-sulphate systems. Cem. Concr. Res..

[37] Pourchez J., Grosseau P., Guyonnet R., Ruot B. (2006). HEC influence on cement hydration measured by conductometry. Cem. Concr. Res..

[38] Ou Z.H., Ma B.G., Jian S.W. (2012). Influence of cellulose ethers molecular parameters on hydration kinetics of Portland cement at early ages. Constr. Build. Mater..

[39] Pourchez J., Peschard A., Grosseau P., Guyonnet R., Guilhot B., Vallée F. (2006). HPMC and HEMC influence on cement hydration. Cem. Concr. Res..

[40] Xu K., Yang J., He H.J., Wei J.J., Zhu Y.P. (2025). Influences of Additives on the Rheological Properties of Cement Composites: A Review of Material Impacts. Materials.

[41] Pourchez J., Govin A., Grosseau P., Guyonnet R., Guilhot B., Ruot B. (2006). Alkaline stability of cellulose ethers and impact of their degradation products on cement hydration. Cem. Concr. Res..

[42] Pourchez J., Grosseau P., Ruot B. (2010). Changes in C3S hydration in the presence of cellulose ethers. Cem. Concr. Res..

[43] Chen W.T., Zhou Y., Yu Q.J., Zhan B.G., Li W.H., Xiong C.C., Chen S.J., Cheng L.Q., Zheng Y.Z.Z. (2024). Microscopic thickening mechanisms of hydroxypropyl methyl cellulose ether anti-washout admixture and its impact on cementitious material rheology and anti-dispersal performance. J. Build. Eng..

[44] Phan T.H., Chaouche M., Moranville M. (2006). Influence of organic admixtures on the rheological behavior of cement pastes. Cem. Concr. Res..

[45] Schmidt W., Brouwers J., Kühne H.-C., Meng B. (2010). Effects of superplasticizer and viscosity-modifying agent on fresh concrete performance of SCC at varied ambient temperatures. Design, Production and Placement of Self-Consolidating Concrete.

[46] Zhang K., Kong D.Y., Schmidt W., Mezhov A. (2025). Influence of hydroxypropylation of starch on rheological properties of cement paste modified by superplasticizers. Constr. Build. Mater..

[47] Ali N., Soliman A.M. (2025). Influence of viscosity modifier addition methods on the rheological behavior of alkali-activated slag systems. Constr. Build. Mater..

[48] Cappellari M., Daubresse A., Chaouche M. (2013). Influence of organic thickening admixtures on the rheological properties of mortars: Relationship with water-retention. Constr. Build. Mater..

[49] Paiva H., Silva L.M., Labrincha J.A., Ferreira V.M. (2006). Effects of a water-retaining agent on the rheological behavior of a single-coat render mortar. Cem. Concr. Res..

[50] Bouras R., Kaci A., Chaouche M. (2012). Influence of viscosity modifying admixtures on the rheological behavior of cement and mortar pastes. Korea-Aust. Rheol. J..

[51] Xiao J., Hou S., Duan Z., Zou S. (2023). Rheology of 3D printable concrete prepared by secondary mixing of ready-mix concrete. Cem. Concr. Comp..

[52] Figueiredo S.C., Rodríguez C.R., Ahmed Z.Y., Bos D.H., Xu Y., Salet T.M., Çopuroğlu O., Schlangen E., Bos F.P. (2019). An approach to develop printable strain hardening cementitious composites. Mater. Des..

[53] Assaad J., Khayat K.H., Mesbah H. (2003). Assessment of thixotropy of flowable and self-consolidating concrete. Mater. J..

[54] Assaad J.J., Khayat K.H. (2006). Effect of viscosity-enhancing admixtures on formwork pressure and thixotropy of self-consolidating concrete. ACI Mater. J..

[55] Marchon D., Kawashima S., Bessaies-Bey H., Mantellato S., Ng S. (2018). Hydration and rheology control of concrete for digital fabrication: Potential admixtures and cement chemistry. Cem. Concr. Res..

[56] Bessaies-Bey H., Baumann R., Schmitz M., Radler M., Roussel N. (2015). Effect of polyacrylamide on rheology of fresh cement pastes. Cem. Concr. Res..

[57] Brumaud C., Bessaies-Bey H., Mohler C., Baumann R., Schmitz M., Radler M., Roussel N. (2013). Cellulose ethers and water retention. Cem. Concr. Res..

[58] Liu Q., Jiang Q., Huang M., Xin J., Chen P., Wu S. (2022). Modifying effect of anionic polyacrylamide dose for cement-based 3DP materials: Printability and mechanical performance tests. Constr. Build. Mater..

[59] Saric-Coric M., Khayat K.H., Tagnit-Hamou A. (2003). Performance characteristics of cement grouts made with various combinations of high-range water reducer and cellulose-based viscosity modifier. Cem. Concr. Res..

[60] Sonebi M., Perrot A. (2019). Effect of mix proportions on rheology and permeability of cement grouts containing viscosity modifying admixture. Constr. Build. Mater..

[61] Leemann A., Winnefeld F. (2007). The effect of viscosity modifying agents on mortar and concrete. Cem. Concr. Comp..

[62] Zhang L.-M., Kong T. (2006). Aqueous polysaccharide blends based on hydroxypropyl guar gum and carboxymethyl cellulose: Synergistic viscosity and thixotropic properties. Colloid Polym. Sci..

[63] Ma S., Li W., Zhang S., Ge D., Yu J., Shen X. (2015). Influence of sodium gluconate on the performance and hydration of Portland cement. Constr. Build. Mater..

[64] Govin A., Bartholin M.-C., Biasotti B., Giudici M., Langella V., Grosseau P. (2016). Modification of water retention and rheological properties of fresh state cement-based mortars by guar gum derivatives. Constr. Build. Mater..

[65] Sun Z.Y., Li Y.J., Ming X., Liu Q., Li Z.J., Chen B.M. (2023). Yield stress of in situ polymerization modified cement paste. Cem. Concr. Res..

[66] Das S., Lee J., Wei J.Q. (2026). Tailoring ultra-high performance concrete for additive manufacturing: Synergistic roles of superplasticizer and viscosity-modifying admixture in printability and performance. J. Build. Eng..

[67] Teng L., Wei J.J., Khayat K.H., Assaad J.J. (2023). Effect of competitive adsorption between specialty admixtures and superplasticizer on structural build-up and hardened property of mortar phase of ultra-high-performance concrete. Cem. Concr. Comp..

[68] González-Aviña J.V., Hosseinpoor M., Yahia A., Durán-Herrera A. (2024). Synergistic effects of superplasticizers and biopolymer-based viscosity-modifying admixtures on the rheology of cement-based systems. Cem. Concr. Comp..

[69] Mezhov A., Goncharov A., Zhutovsky S., Kovler K., Diesendruck C.E. (2025). The Impact of DNA as a Chemical Admixture for Cementitious Materials. Adv. Mater. Technol..

[70] Bai Y.J., Bai Y., Su H., Li J., Hu B.W. (2024). Anti-dispersion, rheological, and mechanical properties of GBFS/FA geopolymer for underwater engineering applications. Constr. Build. Mater..

[71] Domone P. (1998). The Slump Flow Test for High-Workability Concrete 11Communicated by C.D. Pomeroy. Cem. Concr. Res..

[72] Chen B., Liu J. (2005). Contribution of hybrid fibers on the properties of the high-strength lightweight concrete having good workability. Cem. Concr. Res..

[73] Xie Z., Yuan Q., Yao H., Liu Y., Zhang S., Tian Y. (2023). Understanding the impact of polyacrylamide molecular weight on the workability of cement paste. Cem. Concr. Comp..

[74] Lachemi M., Hossain K.M.A., Lambros V., Nkinamubanzi P.C., Bouzoubaâ N. (2004). Performance of new viscosity modifying admixtures in enhancing the rheological properties of cement paste. Cem. Concr. Res..

[75] Łaźniewska-Piekarczyk B. (2013). Effect of viscosity type modifying admixture on porosity, compressive strength and water penetration of high performance self-compacting concrete. Constr. Build. Mater..

[76] Chen Y., Chaves Figueiredo S., Li Z., Chang Z., Jansen K., Çopuroğlu O., Schlangen E. (2020). Improving printability of limestone-calcined clay-based cementitious materials by using viscosity-modifying admixture. Cem. Concr. Res..

[77] Marquez A., Varela H., Barluenga G. (2026). Influence of rheology modifying admixtures on the buildability of 3D printing cement-based mortars. J. Sustain. Cem.-Based Mater..

[78] Kurniati E.O., Kim H.J. (2025). Enhancing the printability of 3D printing limestone calcined clay cement using hydroxyethyl cellulose admixture and silica fume. Constr. Build. Mater..

[79] Kaya E., Ciza B., Yalçinkaya Ç., Felekoglu B., Yazici H., Çopuroglu O. (2024). Effect of hydroxypropyl methylcellulose and aggregate volume on fresh and hardened properties of 3D printable concrete. Constr. Build. Mater..

[80] Zhang Y., Zhang Z.H., Li X.R., Li W.F., Shen X.D., Wang H. (2018). Effect of welan gum on the hydration and hardening of Portland cement. J. Therm. Anal. Calorim..

[81] Poinot T., Govin A., Grosseau P. (2013). Impact of hydroxypropylguars on the early age hydration of Portland cement. Cem. Concr. Res..

[82] Yuan Q., Xie Z., Yao H., Fan M., Huang T. (2022). Comparative study on the early properties of cement modified with different ionic polyacrylamides. Constr. Build. Mater..

[83] Sun Z., Xu Q. (2008). Micromechanical analysis of polyacrylamide-modified concrete for improving strengths. Mater. Sci. Eng. A.

[84] Chaves Figueiredo S., Çopuroğlu O., Schlangen E. (2019). Effect of viscosity modifier admixture on Portland cement paste hydration and microstructure. Constr. Build. Mater..

[85] Ciobanu C., Lazau I., Pacurariu C. (2013). Investigation regarding the effect of viscosity modifying admixtures upon the Portland cement hydration using thermal analysis. J. Therm. Anal. Calorim..

[86] Spychal E., Czapik P. (2020). The Influence of HEMC on Cement and Cement-Lime Composites Setting Processes. Materials.

[87] Kubissa W., Wilińska I., Jaskulski R. (2022). Study on the effect of VMA admixture for concrete cured under different conditions on air permeability and sorptivity. Constr. Build. Mater..

[88] Knapen E., Van Gemert D. (2009). Cement hydration and microstructure formation in the presence of water-soluble polymers. Cem. Concr. Res..

[89] Hansen W. (1960). Actions of calcium sulfate and admixtures in Portland cement pastes. Symposium on Effect of Water-Reducing Admixtures and Set-Retarding Admixtures on Properties of Concrete.

[90] Thomas N., Double D. (1981). Calcium and silicon concentrations in solution during the early hydration of Portland cement and tricalcium silicate. Cem. Concr. Res..

[91] Banfill P., Saunders D. (1986). The relationship between the sorption of organic compounds on cement and the retardation of hydration. Cem. Concr. Res..

[92] Silva D., Roman H., John V. Effects of EVA and HEC polymers on the Portland cement hydration. Proceeding of the 11th International Congress on Polymers in Concrete.

[93] Young J.F. (1972). A review of the mechanisms of set-retardation in portland cement pastes containing organic admixtures. Cem. Concr. Res..

[94] Bhattacherjee S., Jain S., Santhanam M. (2024). Investigating the Interaction of Limestone Calcined Clay and OPC-Based Systems with a Methyl Hydroxyethyl Cellulose-Based Viscosity Modifier Used for 3D Printable Concrete. J. Mater. Civ. Eng..

[95] Afridi M.U.K., Ohama Y., Zabar Iqbal M., Demura K. (1990). Morphology of Ca(OH)_2_ in polymer-modified mortars and effect of freezing and thawing action on its stability. Cem. Concr. Comp..

[96] Zhang M., Chang J. (2010). Surfactant-assisted sonochemical synthesis of hollow calcium silicate hydrate (CSH) microspheres for drug delivery. Ultrason. Sonochem..

[97] Zhang H., Wang P., Li W., She W. (2019). Acceleration effect of synthesised calcium silicate hydrate with different morphologies and Ca/Si on cement hydration. Adv. Cem. Res..

[98] Zhou Y., Orozco C.A., Duque-Redondo E., Manzano H., Geng G., Feng P., Monteiro P.J.M., Miao C. (2019). Modification of poly(ethylene glycol) on the microstructure and mechanical properties of calcium silicate hydrates. Cem. Concr. Res..

[99] Zhi F.F., Yang J.Z., Tang X.D., Bai Z.P., Wang L., Gu Y., Zhang L., Jin W.Z., Yang G.H., Chu H.Q. (2025). Effect of polyacrylamide on the hydration product of portlandite in hardened cement paste. J. Therm. Anal. Calorim..

[100] Zhao L.X., Feng P., Hong J.X., Liu Q., Geng G.Q. (2024). Improved Low-field NMR porosity characterization of cementitious materials containing water-soluble organic admixtures. Cem. Concr. Comp..

[101] Silva B.A., Ferreira Pinto A.P., Gomes A., Candeias A. (2020). Impact of a viscosity-modifying admixture on the properties of lime mortars. J. Build. Eng..

[102] Zhao L., Feng P., Shao L., Ye S., Liu X. (2021). Using viscosity modifying admixture to reduce diffusion in cement-based materials: Effect of molecular mass. Constr. Build. Mater..

[103] Pourchez J., Ruot B., Debayle J., Pourchez E., Grosseau P. (2010). Some aspects of cellulose ethers influence on water transport and porous structure of cement-based materials. Cem. Concr. Res..

[104] Wang S., Zhang G., Wang Z., Huang T., Wang P. (2022). Evolutions in the properties and microstructure of cement mortars containing hydroxyethyl methyl cellulose after controlling the air content. Cem. Concr. Comp..

[105] Li J., Wang R., Li L. (2021). Influence of cellulose ethers structure on mechanical strength of calcium sulphoaluminate cement mortar. Constr. Build. Mater..

[106] Bahranifard Z., Farshchi Tabrizi F., Vosoughi A.R. (2019). An investigation on the effect of styrene-butyl acrylate copolymer latex to improve the properties of polymer modified concrete. Constr. Build. Mater..

[107] Knapen E., Van Gemert D. (2015). Polymer film formation in cement mortars modified with water-soluble polymers. Cem. Concr. Comp..

[108] Wang M., Wang R., Yao H., Farhan S., Zheng S., Wang Z., Du C., Jiang H. (2016). Research on the mechanism of polymer latex modified cement. Constr. Build. Mater..

[109] Wang Y., Liu Q. (2021). Investigation on fundamental properties and chemical characterization of water-soluble epoxy resin modified cement grout. Constr. Build. Mater..

[110] Aliabdo A.A.E., Abd_Elmoaty A.E.M. (2012). Experimental investigation on the properties of polymer modified SCC. Constr. Build. Mater..

[111] Zhi F., Jiang Y., Guo M.-Z., Jin W., Yan X., Zhu P., Jiang L. (2022). Effect of polyacrylamide on the carbonation behavior of cement paste. Cem. Concr. Res..

[112] Omikrine Metalssi O., Aït-Mokhtar A., Ruot B. (2014). Influence of cellulose ether on hydration and carbonation kinetics of mortars. Cem. Concr. Comp..

[113] Silva B.A., Ferreira Pinto A.P., Gomes A., Candeias A. (2021). Short- and long-term properties of lime mortars with water-reducers and a viscosity-modifier. J. Build. Eng..

[114] Duran-Herrera A., Mendoza-Rangel J.M., De-Los-Santos E.U., Vazquez F., Valdez P., Bentz D.P. (2015). Accelerated and natural carbonation of concretes with internal curing and shrinkage/viscosity modifiers. Mater. Struct..

[115] Bentz D.P., Snyder K.A., Peltz M.A., Obla K., Kim H. (2013). Viscosity Modifiers to Enhance Concrete Performance. ACI Mater. J..

[116] Bentz D.P., Snyder K.A., Peltz M.A. (2010). Doubling the service life of concrete structures. II: Performance of nanoscale viscosity modifiers in mortars. Cem. Concr. Comp..

[117] Bentz D.P., Davis J.M., Peltz M.A., Snyder K.A. (2014). Influence of internal curing and viscosity modifiers on resistance to sulfate attack. Mater. Struct..

[118] Singh N.K., Mishra P.C., Singh V.K., Narang K.K. (2003). Effects of hydroxyethyl cellulose and oxalic acid on the properties of cement. Cem. Concr. Res..

[119] Khayat K.H. (1996). Effects of antiwashout admixtures on properties of hardened concrete. ACI Mater. J..

[120] Shadkam H.R., Dadsetan S., Tadayon M., Sanchez L.F.M., Zakeri J.A. (2017). An investigation of the effects of limestone powder and Viscosity Modifying Agent in durability related parameters of self-consolidating concrete (SCC). Constr. Build. Mater..

[121] Wang S., Zhang G., Wang Z., Luo S., Huang T., Wu M. (2021). Long-term performance and hydration of cement mortars with hydroxyethyl methyl cellulose cured at 5℃ low temperature. Constr. Build. Mater..

[122] Izaguirre A., Lanas J., Alvarez J.I. (2011). Characterization of aerial lime-based mortars modified by the addition of two different water-retaining agents. Cem. Concr. Comp..

[123] Bílek V., Kalina L., Novotný R. (2018). Polyethylene glycol molecular weight as an important parameter affecting drying shrinkage and hydration of alkali-activated slag mortars and pastes. Constr. Build. Mater..

[124] Ye H., Fu C., Lei A. (2020). Mitigating shrinkage of alkali-activated slag by polypropylene glycol with different molecular weights. Constr. Build. Mater..

[125] Yu F., Lou Z., Yan N. (2021). Effect of the compounding of an antifoaming agent and a viscosity modifying agent on the frost resistance of mold bag concrete. Constr. Build. Mater..

[126] Łaźniewska-Piekarczyk B. (2013). The type of air-entraining and viscosity modifying admixtures and porosity and frost durability of high performance self-compacting concrete. Constr. Build. Mater..

[127] Yamato T., Soeda M. (1991). Freezing and thawing resistance of anti-washout concrete under water. Spec. Publ..

[128] Zhi F.F., Yang G.H., Zhu P.F., Gu Y., Song Z.J., Chu H.Q., Jiang L.H. (2026). Influence of polyacrylamide on the chloride transport in cement pastes. J. Build. Eng..

[129] Zhi F.F., Jiang Y., Li W.W., Yang G.H., Zhu P.F., Chu H.Q., Jiang L.H. (2025). Effect of cellulose ethers on the chloride transport in cement pastes. Constr. Build. Mater..

[130] Zhi F.F., Yang J.Z., Yang G.H., Zhang L., Li W.W., Jiang L.H. (2024). Investigation on the calcium leaching behaviors of cellulose ethers containing cement pastes. Cem. Concr. Comp..

[131] Zhao L.X., Feng P., Shen X.Y., Rong H., Miao C.W., Geng G.Q. (2024). Mitigating chloride attack in cementitious materials without compromising other properties via the use of viscosity modifying admixture. Cem. Concr. Comp..

[132] Zhao L.X., Feng P., Chen C., Liu Q., Geng G.Q. (2024). A chloride diffusion model for cementitious material with pore-solution viscosity enhancement. J. Build. Eng..

[133] Zhi F.F., Tang X.D., Wang L., Bai Z.P., Na B.B., Zhang L., Yang G.H., Jiang L.H. (2025). Influence of Cellulose Ethers on the Carbonation Behavior of Cement Pastes. J. Mater. Civ. Eng..

[134] de Souza A.M., de Carvalho J.M.F., Silva G.M., Pedroti L.G., Silva G.J.B., Peixoto R.A.F. (2025). The influence of LC3 on the rheology of cementitious matrices: A systematic review of key rheological impact characteristics. Mater. Struct..

[135] Li C., Miao L., You Q., Hu S., Fang H. (2018). Effects of viscosity modifying admixture (VMA) on workability and compressive strength of structural EPS concrete. Constr. Build. Mater..

[136] Lu H., Dai B., Li C., Wei H., Wang J. (2025). Flocculation Mechanism and Microscopic Statics Analysis of Polyacrylamide Gel in Underwater Cement Slurry. Gels.

[137] Zou S., Sun Z., Lu Z., Yang H., Li H., Fan S., Deng X., Yang J. (2024). Fresh and hardened properties of cement paste under the synergistic influence of polycarboxylate superplasticizer and anionic polyacrylamide. Case Stud. Constr. Mater..

[138] Zhao L., Feng P., Ye S., Wang H., He J., Yuan S. (2022). Exploration of offsetting the negative effects of polyethylene glycol admixture on mortar performance: Using diethanol-isopropanolamine. Constr. Build. Mater..

[139] Zhao L., Feng P., Ye S., Liu X., Wang H. (2021). Effect of polyethylene glycol on chloride binding in mortar. Constr. Build. Mater..

[140] Sarbapalli D., Dhabalia Y., Sarkar K., Bhattacharjee B. (2017). Application of SAP and PEG as curing agents for ordinary cement-based systems: Impact on the early age properties of paste and mortar with water-to-cement ratio of 0.4 and above. Eur. J. Environ. Civ. Eng..

[141] Dhir R.K., Hewlett P.C., Lota J.S., Dyer T.D. (1994). An investigation into the feasibility of formulating self-cure concrete. Mater. Struct..

[142] Teng L., Jin M., Du J., Khayat K.H. (2024). Synergetic effect of viscosity modifying admixtures and polycarboxylate ether superplasticizer on key characteristics of thixotropic UHPC for bonded bridge deck overlay rehabilitation. Case Stud. Constr. Mater..

[143] Kang Y.Y., Yu C., Zhang Z.D., Jia L.T., Wang X.G., Banthia N., Zhang Y.M., Jia Z.J. (2026). The coupling effect of viscosity modifying agents and printing process on the air-void structure formation of 3D printed air-entrained concrete. Cem. Concr. Comp..

[144] Biasotti B., Giudici M., Langella V., Pfeiffer U. Highly substituted hydroxypropylguar: A strong contribution to construction chemistry. Proceedings of the Third International Drymix Mortar Conference.

[145] Li P.P., Zhang C., Hu M.M., Yu Y.J., Liu M., Xia X.J., Cao J., Cheng Y., Guo J.T. (2024). Nanosilica interface graft copolymer for improving the suspension stability and filtration performance of oil-well cement slurry. J. Mol. Liq..

